# Identification of Novel Key Molecular Signatures in the Pathogenesis of Experimental Diabetic Kidney Disease

**DOI:** 10.3389/fendo.2022.843721

**Published:** 2022-03-30

**Authors:** Meng Diao, Yimu Wu, Jialu Yang, Caiying Liu, Jinyuan Xu, Hongchao Jin, Juan Wang, Jieping Zhang, Furong Gao, Caixia Jin, Haibin Tian, Jingying Xu, Qingjian Ou, Ying Li, Guotong Xu, Lixia Lu

**Affiliations:** ^1^ Department of Ophthalmology, Shanghai Tongji Hospital of Tongji University, Laboratory of Clinical Visual Science of Tongji Eye Institute, School of Medicine, Tongji University, Shanghai, China; ^2^ Department of Biochemistry and Molecular Biology, Tongji University School of Medicine, Shanghai, China; ^3^ Business School and Science School, University of Auckland, Auckland, New Zealand; ^4^ Department of Human Genetics, Tongji University School of Medicine, Shanghai, China; ^5^ Department of Pharmacology, Tongji University School of Medicine, Shanghai, China; ^6^ Department of Endocrinology, Tongji Hospital of Tongji University, Shanghai, China

**Keywords:** diabetic kidney disease, bioinformatics, ketone body metabolism, Mpv17l, HMGCS2, BDH1

## Abstract

Diabetic kidney disease (DKD) is a long-term major microvascular complication of uncontrolled hyperglycemia and one of the leading causes of end-stage renal disease (ESDR). The pathogenesis of DKD has not been fully elucidated, and effective therapy to completely halt DKD progression to ESDR is lacking. This study aimed to identify critical molecular signatures and develop novel therapeutic targets for DKD. This study enrolled 10 datasets consisting of 93 renal samples from the National Center of Biotechnology Information (NCBI) Gene Expression Omnibus (GEO). Networkanalyst, Enrichr, STRING, and Cytoscape were used to conduct the differentially expressed genes (DEGs) analysis, pathway enrichment analysis, protein-protein interaction (PPI) network construction, and hub gene screening. The shared DEGs of type 1 diabetic kidney disease (T1DKD) and type 2 diabetic kidney disease (T2DKD) datasets were performed to identify the shared vital pathways and hub genes. Strepotozocin-induced Type 1 diabetes mellitus (T1DM) rat model was prepared, followed by hematoxylin & eosin (HE) staining, and Oil Red O staining to observe the lipid-related morphological changes. The quantitative reverse transcription-polymerase chain reaction (qRT-PCR) was conducted to validate the key DEGs of interest from a meta-analysis in the T1DKD rat. Using meta-analysis, 305 shared DEGs were obtained. Among the top 5 shared DEGs, Tmem43, Mpv17l, and Slco1a1, have not been reported relevant to DKD. Ketone body metabolism ranked in the top 1 in the KEGG enrichment analysis. Coasy, Idi1, Fads2, Acsl3, Oxct1, and Bdh1, as the top 10 down-regulated hub genes, were first identified to be involved in DKD. The qRT-PCR verification results of the novel hub genes were mostly consistent with the meta-analysis. The positive Oil Red O staining showed that the steatosis appeared in tubuloepithelial cells at 6 w after DM onset. Taken together, abnormal ketone body metabolism may be the key factor in the progression of DKD. Targeting metabolic abnormalities of ketone bodies may represent a novel therapeutic strategy for DKD. These identified novel molecular signatures in DKD merit further clinical investigation.

## Introduction

The prevalence of diabetes and its related complications are increasing significantly globally. Diabetic kidney disease (DKD), a devastating long-term major microvascular complication of uncontrolled hyperglycemia, affects a large population worldwide. The prevalence of diabetes is projected to reach 578 million cases by 2030 in the world, 30 to 40% of which develop DKD (1). DKD is one of the principal causes of end-stage renal disease (ESDR) worldwide, and the disease progression contributes to irreversible damage to the kidney, impaired quality of life, and premature death (2–4). Although extensive studies focus on the DKD mechanism and oxidative stress, end-products of glycation, autophagy, and apoptosis have been identified to be involved in the pathogenesis of DKD, the exact mechanism of DKD remains to be elucidated (5–8). Currently, the effective therapy to completely halt DKD progression to ESDR is lacking.

Bioinformatics is a new field of biological research aiming to synthesize mathematical, statistical, and computational methods to process biological data. Many public repositories, such as Gene Expression Omnibus (GEO) and ArrayExpress, provide an enormous quantity of data generated by genomic sequencing and microarray chips and comprehensive analyses can be performed by integrating multiple studies to achieve biological understanding. Integrative bioinformatics analysis with larger sample sizes and more minor potential biases is superior to finding new molecular signatures (9). Different bioinformatics analyses for DKD have been reported (10–18). The datasets involved in those studies are either from type 1 diabetic kidney disease (T1DKD) (10–13) or type 2 kidney disease (T2DKD) samples (14–16). Some genes, such as connective tissue growth factor (CTGF) (10), complement 3 (C3) (12), complement 5 (C5) (12), cyclin b2 (Ccnb2) (14), and nuclear receptor subfamily 1 group I member 2 (Nr1i2) (14), have been identified to be molecular signatures and showed great potential as therapeutic targets. In 2020, systematic integrated analysis of genetic and epigenetic variation in DKD patients was reported, and functional annotation suggested the role of inflammation, specifically, apoptotic cell clearance and complement activation in kidney disease development (17). More recently, Gao et al. performed integrative bioinformatics analysis of 3 human datasets associated with early T2DKD (pathologic stages I-III), glomerular DKD, and tubular DKD, respectively, and identified 7 candidate genes (SPARC, POSTN, LUM, KNG1, FN1, VCAN, PTPRO) significantly associated with the progression of DKD (18).

The accelerator hypothesis for diabetes is emerging, which argues that type 1 diabetes mellitus (T1DM) and type 2 diabetes mellitus (T2DM) are the same disorder of insulin resistance set against different genetic backgrounds (19). Based on that, we proposed that there should be key shared pathways and potential targets involved in the pathogenesis of both T1DKD and T2DKD. Unlike previous studies that focused on a single type of DKD, the present study enrolled more datasets of T1DKD and T2DKD, which were integrated with suitable meta-analysis to deeply mine shared molecular mechanism underlying T1DKD and T2DKD. The shared DEGs for T1DKD and T2DKD were used to perform the pathway enrichment analysis and hub gene screening. The study aims to identify novel molecular signatures and therapeutic targets for DKD. Understanding the crucial pathways in DKD could provide a new vision into DKD mechanism study and facilitate the development of novel therapeutic strategies.

## Materials and Methods

### Data Collection

Datasets related to rodent T1DKD and T2DKD were obtained from the National Center of Biotechnology Information (NCBI) GEO. **Table 1** showed the detailed information of datasets, and the inclusion-exclusion criteria are described as followed: (i) all samples are kidney tissues, (ii)datasets should include the control groups and experimental groups, and (iii) the datasets should have an appropriate sample size (the sample size of the experimental group is greater than 2, and the sample size of the control group is greater than 2).

**Table 1:** The detailed information of datasets used in Meta-analysis.

Type	Datasets	Platforms	species	samples	Models	References	Experimental group	Control group	Year
	GSE7253	Affymetrix Rat Genome 230 2.0 Array	Rat	Renal cortical tissues	STZ-induced diabetic (65mg/kg) for 6 weeks	PMID: 18030501	6	6	2007
	GSE103109	Affymetrix Rat Gene 2.0 ST Array	Rat	Kidney tissue	STZ-induced diabetic (65mg/kg) for 4 weeks	N/A	2	3	2018
**T1DKD**	GSE131221	Agilent-074036 SurePrint G3 RatGE v2 8x60K Microarray G4858A (Probe Name version)	Rat	Renal cortical tissues	STZ-induced diabetic (65mg/kg) for 2 weeks	PMID: 32171449	7	5	2020
	GSE118089	Illumina HiSeq 2500 (Mus musculus)	Mouse	Renal cortical tissues	STZ-induced diabetic (55mg/kg) for 24 weeks	PMID: 31624141	6	6	2019
	GSE107942	Illumina HiSeq 2500 (Mus musculus)	Mouse	Renal cortical tissues	STZ-induced diabetic (55mg/kg) for 10 weeks	PMID: 29490938	6	6	2018
	GSE133598	Agilent-074809 SurePrint G3 MouseGE v2 8x60K Microarray	Mouse	Renal cortical tissues	HFD (4 weeks) and STZ (3 days) induced diabetic for 20 weeks	PMID: 31798904	3	3	2020
	GSE117085	Illumina NextSeq 500 (Rattus norvegicus)	Rat	Renal cortical tissues	ZDF (fa/fa) rats for 20 weeks	N/A	6	6	2019
**T2DKD**	GSE134804	Illumina HiSeq 4000 (Rattus norvegicus)	Rat	Renal cortical tissues	ZSF1 rats for 28 weeks	PMID: 31300612	5	5	2019
	GSE87359	Affymetrix Mouse Gene 2.0 ST Array	Mouse	Kidney tissue	db/db mice for 22 weeks	PMID: 28232950	3	3	2017
	GSE90842	Affymetrix Mouse Gene 2.0 ST Array	Mouse	Kidney tissue	db/db mice for 20 weeks	N/A	3	3	2017

### Data Preprocessing

Based on the platform annotation information, probe IDs from different microarray and sequencing platforms were converted to Entrez gene IDs. The mean gene expression value was obtained for the probe mapping to the same gene. After log2 transformation and normalization, the datasets were subsequently subjected to the well-established ComBat procedures to reduce potential study-specific batch effects, and invalid samples were deleted (20). The results were visually presented through the principal component analysis (PCA).

### Differentially Expressed Genes (DEGs) Analysis

The meta-analysis was performed with Networkanalyst using Fisher’s combined probability test (Fisher’s method) (21). Fisher’s method can combine extreme value probabilities (p-values) from several independent tests bearing upon the same overall hypothesis and evaluate the significance using chi-squared tests. An adjusted p-value less than 0.05 was considered statistically significant to select DEGs.

### Functional Annotation and Enrichment Analysis

The Enrichr database (http://amp.pharm.mssm.edu/Enrichr/) was used to analyze and visualize Gene Ontology (GO) terms and the Kyoto Encyclopedia of Genes and Genomes (KEGG) pathways for functional annotation and pathway enrichment analysis of the DEGs (22). To determine the role of the DEGs in human disease, Enrichr was also used to visualize the clinic databases, including ClinVar and GWAS.

### Protein-Protein Interaction (PPI) Network Construction and Hub Gene Screening

The Search Tool for the Retrieval of Interacting Genes (STRING; http://string-db.org/) database was used to establish the PPI networks (23). The hub genes were identified using the CytoHuba plugin in Cytoscape software (version 3.6.1) and Maximal Clique Centrality (MCC) algorithm was selected to identify hub genes in this study (24, 25).

### T1DM Rat Model Preparation

Male Sprague‐Dawley rats weighing 180 to 200g were purchased from Slaccas (Shanghai, China), which were used to prepare a diabetic rat model by a single intraperitoneal injection of streptozotocin (STZ, 65 mg/kg body wt) in 0.1 mol/l citrate buffer (pH 4.5). Only males were used because female rats were less sensitive to STZ (26). Totally, 42 rats were used in this experiment. All rats were weighed and randomly divided into 5 groups for specific time points, including after DM onset 3d (6 rats), 1w (12 rats), 2w (12 rats), 4w (6 rats), and 6w (6 rats), half as control and half as experimental group. Rats were sacrificed at specified time points above, respectively. Rats receiving an injection of an equal volume of 0.1 mol/l citrate buffer were used as normal controls. According to our previous work (27), the model was successfully established when the blood glucose level was higher than 16.7mmol/L for three consecutive days. Some kidneys of rats were stored in 4% paraformaldehyde (PFA) in phosphate buffer solution (PBS), and the others were stored at -80°C for subsequent experiments.

### HE Staining

Kidney tissues fixed in 4% PFA were dehydrated, embedded in paraffin, and cut into 4 μm thick sections. Sections treated with hematoxylin & eosin (HE) staining kit (Sangon Biotech Co., Ltd.NO. E607318) were observed under a light microscope (Olympus, BX53M) at high magnification.

### Oil Red O Staining of Neutral Fat

The 0.5% (w/w) stock solution of Oil Red O (ORO, Sangon Biotech Co., Ltd. NO. E607319) was prepared by adding ORO to a 98%(v/v) isopropyl solution (Sangon Biotech Co., Ltd NO. A507048), which was then filtered through filter paper. Then the filtrate was added to double distilled water to prepare a 60%(v/v) ORO-isopropyl solution (working solution), and the working solution was filtered for the second time by using a 45μm filter. The sections were first put at room temperature for 10 min and then soaked in isopropyl for pretreatment of about 30 sec. The sections were stained by covering the working solution for 30 min, which were then rinsed with 60% (v/v) isopropyl solution and double distilled water successively. Nuclear staining was performed using Mayer′s hematoxylin (Sangon Biotech Co., Ltd. NO. E607317) for 30 sec. Finally, the sections were rinsed under running tap water until they turned blue and were observed under a light microscope (Olympus, BX53M).

### Quantitative Reverse Transcription Polymerase Chain Reaction (qRT-PCR)

Total RNA was extracted from rat kidney tissue with Trizol (Vazyme Inc., Nanjing, China, R401-01) and quantified by NanoDrop2000/2000c spectrophotometer (Thermo Fisher Scientific, US). The cDNA was synthesized using the reverse transcription kit (Vazyme Inc., Nanjing, China, R101-01). The primers for specific genes were designed by primer design tools, the RealTime PCR Tool in INTEGRATED DNA TECHNOLOGIES (www.idtdna.com/site/order/menu). The primer-BLAST in the NCBI website (https://www.ncbi.nlm.nih.gov/tools/primer-blast/index.cgi) was performed to examine the amplification specificity for primers. qRT-PCR was performed according to the manufacturer’s instructions (Vazyme Inc., Nanjing, China, Q221-01). PCR program was as followed: 95°C for 5 min, 95°C for 10 sec, 55°C for 30 sec, 72°C for 10 sec, 40 cycles. The relative quantification of mRNA levels was calculated based on the 2^−ΔΔCt^ method (28), and ACTB was used as an internal control.

### Statistical Analysis

Results were presented as the mean ± Standard Error of Mean (SEM). Statistical analyses were carried out with SPSS 17.0 statistical software (SPSS, Inc., Chicago, IL, USA). The student’s t‐test was used for the statistical significance analysis. A p-value <0.05 was considered to be statistically significant. qPCR figures were prepared with GraphPad Prism 9 (GraphPad Software, Inc., San Diego, CA, USA).

## Results

### Identification of DEGs of Interest

**Figure 1:**
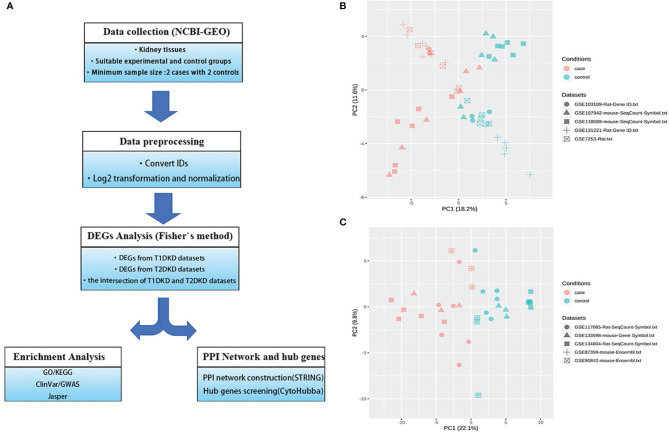
Data collection and processing: **(A)** The Meta-analysis flowchart. The main methods in each stage are described in the corresponding parentheses. **(B, C)** Principal component analysis (PCA) of the combined datasets after batch effect correction. All samples are represented by different symbols with shapes according to their studies and colors based on their experimental conditions. **(B)** T1DKD datasets. **(C)** T2DKD datasets.

The meta-analysis flowchart was shown in **Figure 1A**. Before DEGs analysis, the data should be normalized. The quality reports of the individual datasets were shown in **Figure S1** (before data normalization) and S2 (after data normalization). As shown in **Figures S1K, L**, the samples were tightly clustered mainly according to the original studies. After batch effect correction, the resulting PCA plot demonstrated that the clustering was primarily based on normal control and DKD groups (**Figures 1B, C**), which manifested that the adjusted batch datasets could be used for further analysis.

**Figure 2:**
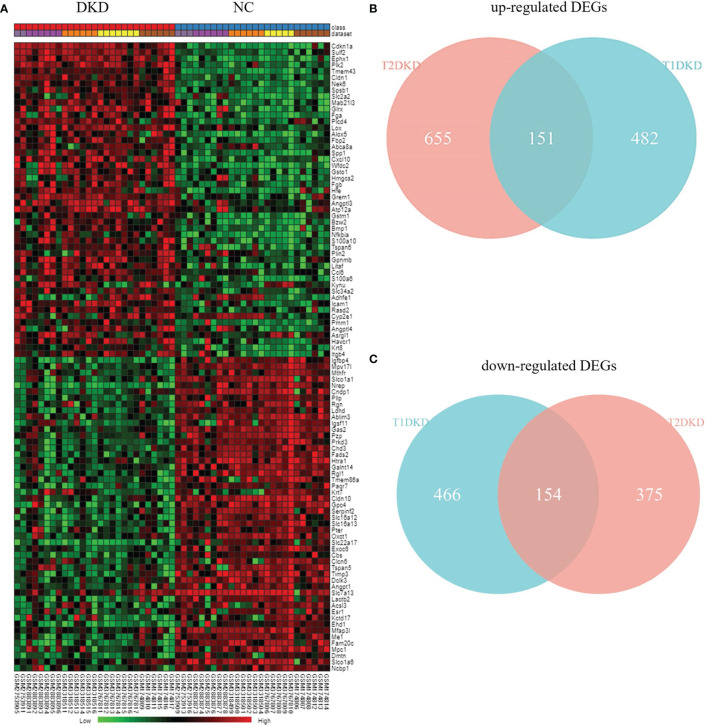
Differentially expressed genes analysis: **(A)** Heatmap of the top 50 shared up- and down-regulated genes identified during meta-analysis. **(B)** Venn diagrams of the shared up-regulated DEGs in TIDKD and T2DKD datasets. **(C)** Venn diagrams of the shared down-regulated DEGs in TIDKD and T2DKD datasets.

According to Fisher’s method, 633 up-regulated genes and 620 down-regulated genes were found in T1DKD datasets. The heatmap of DEGs in T1DKD was shown in **Figure S3A** and the detailed information of the top 5 DEGs were shown in **Supplementary Table 1**. 806 up-regulated genes and 529 down-regulated genes were found in T2DKD datasets. The heatmap of DEGs in T2DKD was shown in **Figure S3B** and the detailed information of the top 5 DEGs were shown in **Supplementary Table 2**. Importantly, 305 shared DEGs were obtained from T1DKD and T2DKD datasets, including 151 up-regulated genes and 154 down-regulated genes. The Venn diagrams of the shared DEGs and the expression heatmaps were shown in **Figure 2**. The detailed information for the most significant shared genes were shown in **Table 2**. The shared up-regulated genes included Cdkn1a, Sulf2, Ephx1, Plk2, etc., and the shared down-regulated genes included Igfbp4, Mthfr, Nrep, etc. Unexpectedly, some shared DEGs (Tmem43, Mpv17l, Slco1a1, etc.) with statistical significance have not been reported before in DKD relevant-related studies.

**Table 2:** Shared DEGs found in meta-analysis.

Ranking	Regulation	Combined P value	Name	Function	Published role in DKD
**1**	Up	0	Cdkn1a	CDK inhibitor and regulate G1 phase of cell cycle	PMID:31545928
**2**	Up	0	Sulf2	plays indispensable protective roles to maintain glomerular integrity	PMID:26764203
**3**	Up	0	Ephx1	promotes hyperglycemia-induced renal endoplasmic reticulum stress, inflammation and fibrosis, and decrease autophagy in DKD	PMID:28757338
**4**	Up	0	Plk2	Role in high D-glucose-induced apoptosis, ROS generation and Inflammation in Podocytes	PMID:28655909
**5**	Up	0	Tmem43	Related to familial arrhythmogenic right ventricular dysplasia type 5	NA
**1**	Down	0	Igfbp4	Bind both insulin-like growth factors (IGFs) and prolong the half-life of the IGFs	PMID:21309053
**2**	Down	0	Mpv17l	Regulate of ROS metabolism and the control of oxidative phosphorylation	NA
**3**	Down	0	Mthfr	catalyzes the conversion of 5,10-CH2-FH4 to 5-CH3-FH4	PMID:16828193
**4**	Down	0	Slco1a1	mediates the Na+-independent transport of organic anions	NA
**5**	Down	0	Nrep	Down-regulate the expression of TGFβ1 and TGFβ2	PMID:32539181

### The Obtainment of Enriched GO Terms and Key Pathways

**Figure 3:**
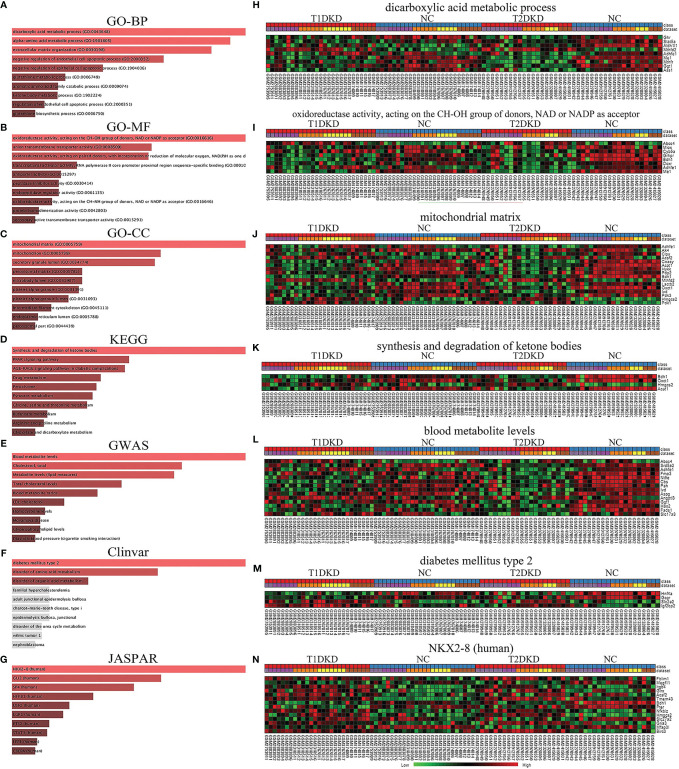
Functional annotation and enrichment analysis of the shared DEGs in T1DKD and T2DKD datasets obtained by meta-analysis: **(A-G)** Functional annotation and enrichment analysis results in Enrichr. **(A)** GO-BP; **(B)** GO-MF; **(C)** GO-CC; **(D)** KEGG; **(E)** GWAS; **(F)** Clinvar; **(G)** JASPAR. **(H–N)** Heatmaps of genes enriched in each top1 term.

Based on the shared DEGs in T1DKD and T2DKD datasets, the GO terms and KEGG pathways were enriched and the top 10 terms were shown in **Figure 3**. In the biological process (BP), the shared DEGs were mainly enriched in the dicarboxylic acid metabolic process and the alpha-amino acid metabolic process in the biological process (**Figure 3A**). In molecular function (MF), DEGs were mainly enriched in oxidoreductase activity (acting on CH-OH group of donors, NAD/NADP as acceptor) (**Figure 3B**). As for the cellular component (CC) group, DEGs were mainly enriched in the mitochondrial matrix (**Figure 3C**). The Venn diagrams of all significant GO common to T2DKD and T1DKD were shown in **Figure S4**. In the KEGG pathway, unexpectedly, synthesis and degradation of ketone bodies ranked first (**Figure 3D**). The heatmap of involved genes, Bdh1, Oxct1, Hmgcs2, and Acat1, were presented in **Figure 3K**. In the results of GWAS analysis, the top 1 was blood metabolite levels and total cholesterol levels were also enriched significantly (**Figure 3E**). Diabetes mellitus type 2 was significantly enriched in the Clinvar database (**Figure 3F**) and NK2 Homeobox 8 (NKX2-8, human) was enriched in the JASPAR database (**Figure 3G**).

### Identification of Hub Genes

**Figure 4:**
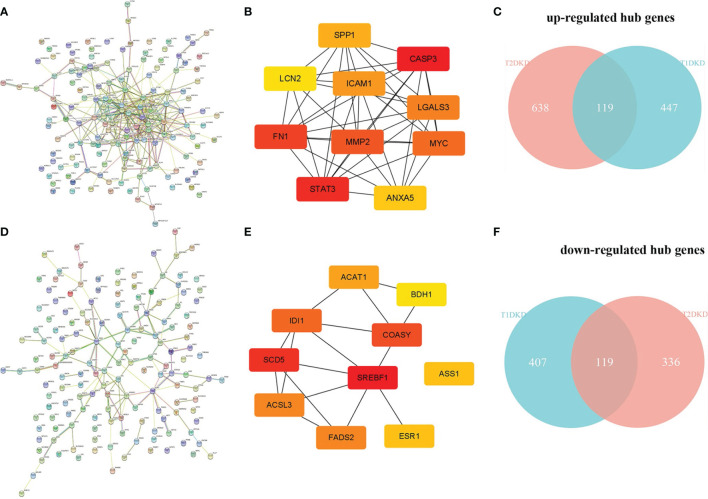
Protein-protein interaction (PPI) network construction and hub gene screening: **(A)** The PPI network constructed with the shared up-regulated DEGs. **(B)** Top 10 up-regulated hub genes. **(C)** Venn diagram of the shared up-regulated hub genes. **(D)** The PPI network constructed with the shared down-regulated DEGs. **(E)** Top 10 down-regulated hub genes. **(F)** Venn diagram of the shared down-regulated hub genes.

The human PPI network based on the shared up-and down-regulated DEGs in T1DKD and T2DKD datasets was constructed (**Figures 4A, D**), and the hub genes screened with Maximal Clique Centrality (MCC) algorithm were shown in **Figures 4B, E**. The Venn diagrams of the shared hub genes were shown in **Figures 4C, F**. The detailed information about the top 10 hub genes were shown in **Table 3**. The top 10 shared up-regulated hub genes included Casp3, stat3, fn1, Mmp2, Myc, Lgals3, Icam1, Spp1, Anxa5, and Lcn2, all of which have been reported to be involved in DKD before. Among the top 10 shared down-regulated hub genes, 8 hub genes, Scd5, Coasy, Idi1, Fads2, Acsl3, Acat1, Ass1, Bdh1, except Srebf1 and Esr1, were novel to DKD study.

**Table 3:** The top 10 shared up-and down-regulated hub genes found in the PPI network.

Ranking	MCC Score	Regulation	Name	Functions in DKD	Published role in DKD
**1**	38005	Up	Casp3	promotes DKD through GSDME-mediated progression to secondary necrosis during apoptosis	PMID: 32104028
**2**	37282	Up	Stat3	treatment with selective STAT3 inhibitor could attenuate kidney injuries in STZ induced diabetic mice	PMID:31699972
**3**	37226	Up	Fn1	up-regulated in podocytes by mechanical stress results from glomerular hypertension	PMID:31675484
**4**	36285	Up	Mmp2	mediates endothelial glycocalyx damage and albuminuria	PMID: 32037077
**5**	27587	Up	Myc	participates in high glucose-mediated endothelial inflammation in DKD	PMID: 35085772
**6**	26686	Up	Lgals3	Galectin-3–mediated AGE-receptor pathway is operating *in vivo* to confer protection toward AGE-induced tissue injury.	PMID:12874444
**7**	26466	Up	Icam1	Icam1 binding activity with LFA-1 could cause kidney tubular and glomeruli injury.	PMID:23346076
**8**	21761	Up	Spp1	enhances glomerular damage, likely through the expression of TGF-beta.	PMID:20130530
**9**	16734	Up	Anxa5	an early marker of renal cell apoptosis.	PMID: 25661914
**10**	10202	Up	Lcn2	an early biomarker of acute kidney injury	PMID: 19148153
**1**	22	Down	Srebf1	was positively correlated with the tubulointerstitial damage score and inflammation.	PMID: 30348828
**2**	19	Down	Scd5	NA	NA
**3**	16	Down	Coasy	NA	NA
**4**	15	Down	Idi1	NA	NA
**5**	13	Down	Fads2	NA	NA
**6**	13	Down	Acsl3	NA	NA
**7**	12	Down	Acat1	NA	NA
**8**	11	Down	Esr1	Intron 1 and intron 2 of the ESR1 gene may contain functionally important regions related to T2DM or ESRD risk	PMID: 17327435
**9**	11	Down	Ass1	NA	NA
**10**	7	Down	Bdh1	NA	NA

### Morphological Changes of Kidney by HE Staining and Oil Red O Staining

**Figure 5:**
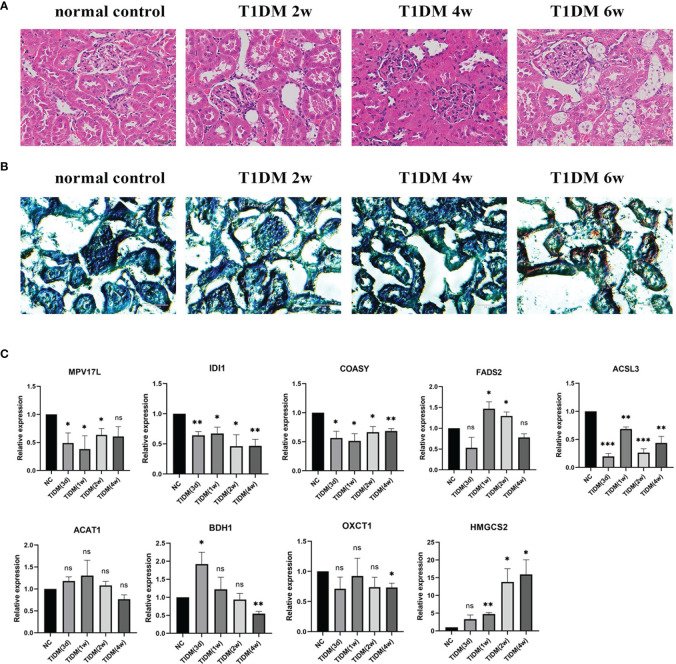
Morphological changes of kidney and qPCR confirmation in T1DM rats: **(A)** Hematoxylin & eosin (HE) staining of renal tissue. **(B)** Oil Red O staining of renal tissue. **(C)** The mRNA expression level of some genes of interest in T1DKD rats. Data are presented as means ± SEM of at least three independent experiments. *p < 0.05, **p < 0.01, ***p < 0.001, and ns, no significance, versus the control with the same treatment (Student’s t-test).

HE staining and Oil Red O staining was performed to examine the morphological changes and lipid droplet deposits in STZ-induced T1DM rats. As shown in **Figure 5A**, in the early stage of DKD (2w), glomeruli in T1DM rats seemed to have no abnormality under light microscopy, characterized with minimal change nephropathy. Tubuloepithelial cell hypertrophy developed at 4w after the onset of T1DM and lipid droplets were observed in epithelial cells of the renal tubules at 6w after the onset of T1DM (**Figure 5B**), suggesting that steatosis appeared in tubuloepithelial cells of T1DM.

### Confirmation of DEGs of Interest at mRNA Level

The qRT-PCR results (**Figure 5C**) showed that in T1DM rats, the mRNA expression levels of Idi1, Acsl3, Coasy, Oxct1, Bdh1, and Mpv17l were significantly down-regulated, and the expression of Fads2 and Hmgcs2 were significantly up-regulated at specific time points. The comparison details of meta-analysis and qRT-PCR results are shown in **Table 4**. The mRNA expression levels of most DEGs of interest in diabetic rats, except Fads2 and Acat1, were consistent with the meta-analysis.

**Table 4:** Meta-analysis details and experimental results of the shared hub genes unpublished relative to DKD and DEGs of interest in T1DKD rats.

Gene Symbol		Idi1	Acsl3	Coasy	Bdh1	Fads2
**Meta-analysis**	CombinedTstat	43.515	71.053	43.705	64.178	84.915
	CombinedPval	3.54E-05	1.60E-09	3.32E-05	2.24E-08	8.15E-12
	LogFC	0.17567	0.30935	0.098564	0.26443	0.1573
	Change in DKD	Decreased	Decreased	Decreased	Decrease	Decreased
**Rats’ experimental results**		Decreased	Decreased	Decreased	Increased (3d)	Increased
					Decreased(4w)	
**Meta-analysis**	**Gene Symbol**	**Acat1**	**Mpv17l**	**Hmgcs2**	**Oxct1**	
	CombinedTstat	64.811	102.29	-73.488	74.656	
	CombinedPval	1.79E-08	0	6.20E-10	4.10E-10	
	LogFC	0.11509	0.57096	-1.1059	0.24781	
	Change in DKD	Decreased	Decreased	Increased	Decreased	
**Rats’ experimental results**		–	Decreased	Increased	Decreased	

## Discussion

Here, we performed an integrative meta-analysis to identify novel vital pathways and molecular signatures for T1DKD and T2DKD, followed by verification in T1DM rats at the transcriptional level. Because T2DKD datasets were derived from a variety of T2DM models, including high-fat diet (HFD) and STZ-induced diabetic rats, ZDF (fa/fa) rats, ZSF1 rats, and db/db mice, experimental verification of meta-analysis results in T2DKD rats was not explored. Nonetheless, this research will provide a new perspective on DKD mechanism research and therapy strategy.

### Novel Molecular Signatures and Potential Therapeutic Targets for DKD

Unlike previous studies, the present study discovered new molecular signatures for the first time, including Mpv17l, Idi1, Coasy, Fads2, Acsl3, Bdh1, and Oxct1. Mpv17l is a transmembrane protein that has previously been linked to peroxisomal reactive oxygen species metabolism and a close homolog of Mpv17, a protein found in the inner mitochondrial membrane protein Mpv17 (29). Mpv17l and HtrA2 complex is critical for mitochondrial oxidative stress sensing and protection from ROS-induced mitochondrial dysfunction. Mpv17 deletion causes proteinuric kidney disease in mice (30). Mpv17l was also linked to the development of early-onset glomerulosclerosis (31) and could modulate antioxidant enzyme expression (32). Mpv17l was found to be down-regulated in both T1DKD and T2DKD rats in a meta-analysis, and the result was confirmed at the transcriptional level in T1DM rats (**Figure 5C**). Oxidative stress has been considered one of the critical mechanisms in the pathogenesis of DKD. Down-regulated Mpv17l in DKD may connect with the increased level of ROS in DKD.

Idi1 is a protein-coding gene that encodes a peroxisomal-localized enzyme called isopentenyl-diphosphate delta isomerase 1. It was reported to catalyze the process of mevalonic acid conversion into farnesyl diphosphate (FPP), and ultimately, cholesterol (33). In 1997, the enzyme was found to be targeted to peroxisomes (34). However, many studies about Idi1 and its product have focused on yeast, plants, etc., and very little research has been done on mammals. Idi1 is closely associated with sterol metabolism and the down-regulation of Idi1 was confirmed at the transcriptional level in T1DM rats in our study (**Figure 5C**). Nevertheless, the current studies have not concluded a link between this gene and the pathogenic process of DKD. Idi1 deserves in-depth study in DKD.

Coasy is a bifunctional protein that carries out the last two steps in the biosynthesis of coenzyme A (CoA) from pantothenic acid (35). Previous studies relevant to Coasy mainly focused on neurodegenerative disease in the central nervous system (36–38). Also, abnormal regulated CoA metabolism in the liver and skeletal muscle likely contributed to the diabetic phenotype of the leptin-deficient mice (39). The down-regulation of Coasy in our results (**Figure 5C**) indicated the possible disruption of CoA synthesis, which might influence the multiple metabolism process, such as sterol, fatty acid, and ketone body in DKD.

The delta-6 desaturase (D6D) enzyme, encoded by the Fads2 gene, is one of the two rate-limiting enzymes that convert the polyunsaturated fatty acid (PUFA) precursors – α-linolenic (ALA, 18:3n-3) and linoleic acid (LA, 18:2n-6) to their individual metabolites, eicosapentaenoic acid/docosahexaenoic acid (EPA/DHA) and arachidonic acid (AA, 20:4n-6). Alterations in the D6D enzyme activity alter fatty acid profiles and are associated with metabolic and inflammatory diseases (40). Fads2 was critical for maintaining body long-chain polyunsaturated fatty acid (LC-PUFA) homeostasis (41). Moreover, LC-PUFA metabolism was associated with the crucial pathways, including the PPAR signaling pathway, spliceosome and protein processing in the endoplasmic reticulum pathway, and candidate kinase targets. The dysregulation of Fads2 may be related to abnormal lipid metabolism in DKD. In our study, the mRNA expression of Fads2 increased in T1DM rats, which was inconsistent with the result in the meta-analysis (**Figure 5C**), probably resulting from different time points of sampling and the small sample size.

Acsl3 is an isozyme of the long-chain fatty-acid-coenzyme A ligase family. It converts free long-chain fatty acids into fatty acyl-CoA esters and plays a crucial role in lipid biosynthesis and fatty acid degradation. Acsl3 is highly expressed in the brain and preferentially utilizes myristate, arachidonate, and eicosapentaenoate as substrates. TNF-α induced up-regulation of Acsl3, and Acsl3 was required for TNF-α-induced lipid droplet formation in endothelial cells exposed to oleic acid, and Acsl3 showed a modest suppression of TNF-α-mediated secretion of PGE2 (42). Acsl3 was shown to promote endogenous fatty acid oxidation (FAO) (43). The down-regulation expression of Acsl3 in the meta-analysis was validated in T1DKD rats using qRT-PCR (**Figure 5C**). We hypothesize that the decreased expression of Acsl3 may lead to an elevated level of FFA and disruption of FAO. Thus, Acsl3 may be considered as a potential therapeutic target for DKD.

### Synthesis and Degradation of Ketone Bodies and Lipid Metabolism

Interestingly, synthesis and degradation of ketone bodies ranked the top 1, followed by the PPAR signal pathway from KEGG pathway enrichment analysis (**Figure 3D**). Although extrahepatic ketogenesis is controversial, the kidney has shown great potential as a ketogenic organ. In 2011, Zhang et al. found that diabetic kidneys exhibited excess ketogenic activity resulting from increased HMGCS2 expression in T2DM (44). Since then, there have been few reports of renal ketogenesis. It is well-accepted that the kidney can excrete and resorb liver-derived ketone bodies from the bloodstream, and beta-hydroxybutyrate (βOHB), the primary form of ketone body, is metabolized by the proximal tubule (45). In 2003, Guh et al. first demonstrated βOHB effected on human proximal tubule cells (HK-2 cells) in terms of cellular growth inhibition and collagen production in a dose-dependent way (46), which may lead to tubulointerstitial fibrosis in DKD. However, in the last few years, more studies focused on the role of ketogenesis in mediating the renal protective effects in diabetes. In 2017, Yi et al. noted that renal ketogenesis could resist oxidation *via* SIRT3 activation in the context of the HFD (47). Ketogenesis is affected by multiple factors, including endocrine regulation, transcriptional regulation, posttranslational modifications, and biochemical regulation (48). As the primary anabolic hormone, insulin suppresses lipolysis in adipose tissue, thus depriving ketogenesis of its substrate (49). PPAR signal pathway, especially PPARα, also plays a role in fatty acid oxidation and ketogenesis (50).

The dysregulation of ketone body metabolism related genes was identified in T1DKD and T2DKD with the down-regulation of Acat1, Oxct1, Bdh1, and the up-regulation of expression of Hmgcs2 by meta-analysis. Hmgcs2, the critical enzyme for ketone body anabolism, condenses acetyl-CoA with acetoacetyl-CoA to form HMG-CoA (51). Acat1 is Acetyl-CoA acetyltransferase that is also involved in ketone body anabolism. Oxct1 is a homodimeric mitochondrial matrix enzyme that plays a central role in extrahepatic ketone body catabolism by catalyzing the reversible transfer of coenzyme A from succinyl-CoA to acetoacetate. Bdh1 catalyzes the interconversion of acetoacetate and βOHB, the two major ketone bodies produced during fatty acid catabolism (52), and Bdh1 was identified as one of the top ten down-regulated hub genes in this study. According to the previous study, βOHB has a primarily anti-inflammatory effect, but high quantities of ketone bodies, notably acetoacetate, may have a pro-inflammatory effect (53). The down-regulation of Bdh1 resulted in acetoacetate accumulation and βOHB decrease in the diabetic kidney, which might contribute to a pro-inflammatory state in DKD. In the present study, the up-regulation expression of Hmgcs2 and the down-regulation expression of Bdh1 and Oxct1 were validated in T1DKD rats by qRT-PCR. Combined with previous studies, we propose an imbalance between the up-regulated ketogenic activity and down-regulated ketolytic activity in diabetic kidneys, which indicated that the diabetic kidney was more likely to work as a ketogenic organ rather than a ketolytic organ. Hmgcs2 and Oxct1, as the key enzymes involved in ketone body metabolism, may be considered potential therapeutic targets. Acat1, one of the top 10 shared down-regulated hub genes, probably has a vital role in both T1DKD and T2DKD. However, the expression of Acat1 in T1DKD rats was not significantly down-regulated compared to the normal group, and its clinic value in the treatment of DKD remains to be elucidated.

In summary, using the meta-analysis approach, for the first time, we identified the novel molecular signatures for DKD, Mpv17l, Idi1, Coasy, Oxct1, Fads2, Bdh1, and Acsl3. Meanwhile, we found that abnormal ketone body metabolism may play a pivotal role in the pathogenesis of DKD. This study deepened our understanding of the molecular mechanism underlying DKD, and targeting metabolic abnormalities of ketone bodies may represent a novel therapeutic strategy for DKD.

## Data Availability Statement

The datasets presented in this study can be found in online repositories. The names of the repository/repositories and accession number(s) can be found in the article/**Supplementary Material**.

## Ethics Statement

The animal study was reviewed and approved by ARVO Statement for the Use of Animals in Ophthalmic and Vision Research and The Guides for the Care and Use of Animals (National Research Council and Tongji University) (Permit Number: TJmed-010-32).

## Author Contributions

LL, YL, and GX conceived, developed, and mentored the project. MD, YW, and JY performed the experiments. JZ, HT, JingX, CJ, FG, and JW provided technical support and analyzed the data. HJ conducted the statistical analysis using R. MD, YW, JY, and LL wrote the manuscript. All authors read and approved the final manuscript.

## Funding

This work was supported by grant obtained from the Ministry of Science and Technology of China (2020YFA0113101) and the Fundamental Research Funds for the Central Universities (22120220009).

## Conflict of Interest

The authors declare that the research was conducted in the absence of any commercial or financial relationships that could be construed as a potential conflict of interest.

## Publisher’s Note

All claims expressed in this article are solely those of the authors and do not necessarily represent those of their affiliated organizations, or those of the publisher, the editors and the reviewers. Any product that may be evaluated in this article, or claim that may be made by its manufacturer, is not guaranteed or endorsed by the publisher.
