# Supplementary material for: Identification of Novel Key Molecular Signatures in the Pathogenesis of Experimental Diabetic Kidney Disease
Source: Front Endocrinol (Lausanne). 2022 Mar 30;13:843721. doi: 10.3389/fendo.2022.843721 (PMC9005898; doi:10.3389/fendo.2022.843721)
Supplement: Supplementary file 1 [file DataSheet_1.docx]

**
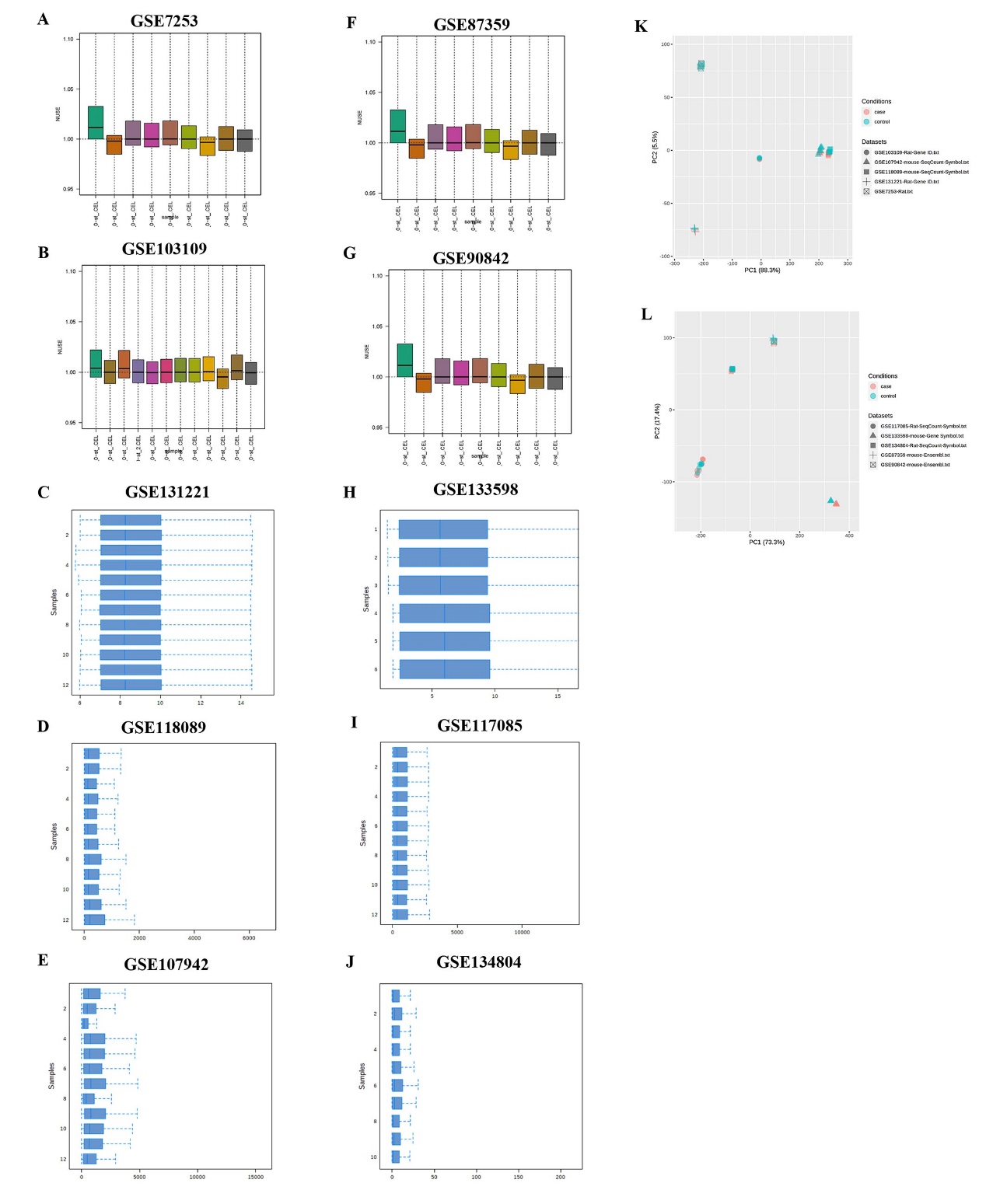
**

**Supplementary Figure 1**

(A-J): Boxplots of 10 datasets before data normalization.

(K-L): Principal component analysis of the combined datasets before batch effect correction. (K) T1DKD datasets. (L)T2DKD datasets.

**
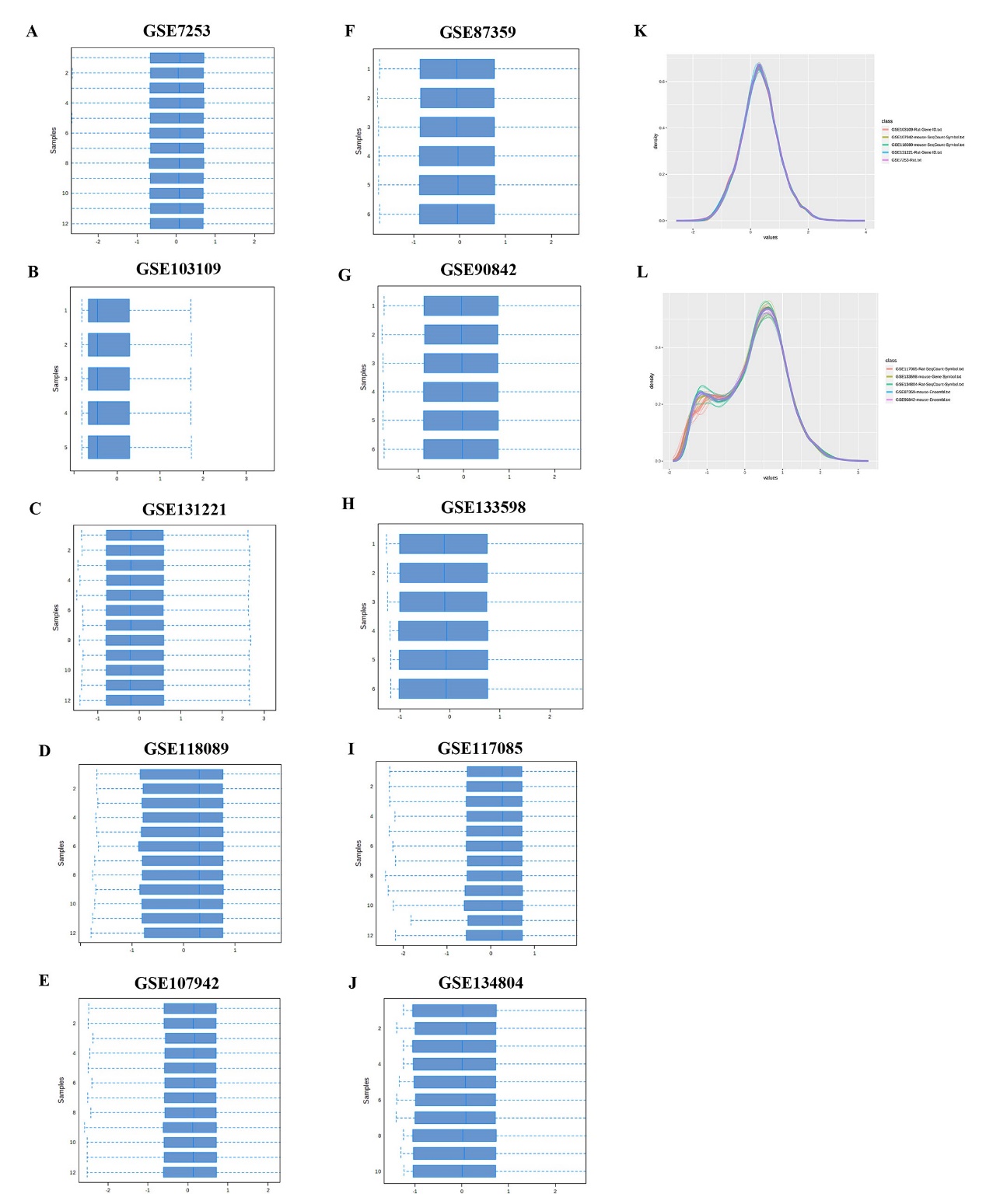
**

**Supplementary Figure 2**

(A-J): Boxplots of 10 datasets after data normalization.

(K-L): Density plots of the combined datasets after batch effect correction. (K) T1DKD datasets. (L)T2DKD datasets.

**
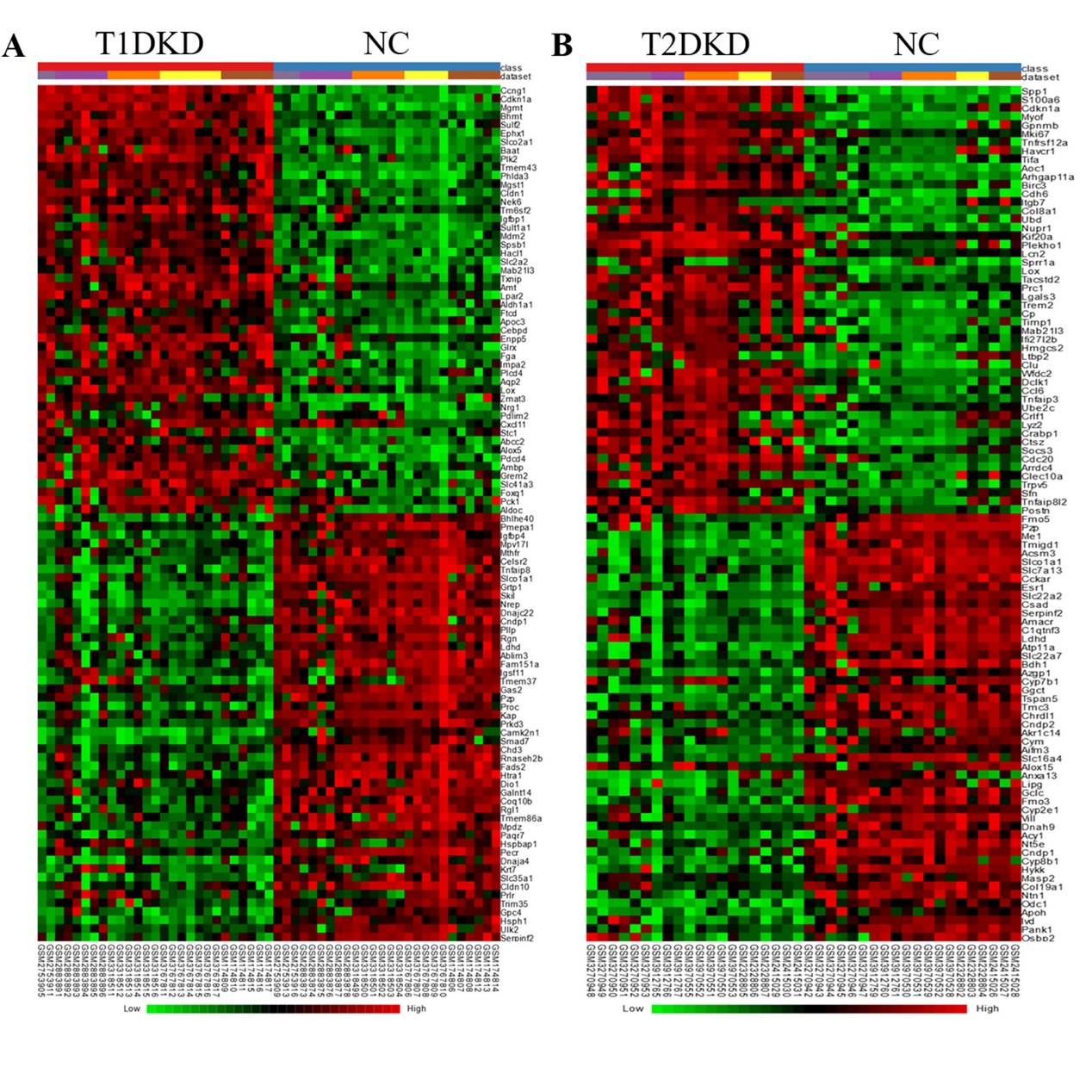
**

**Supplementary Figure 3 Heatmap of the top 50 up- and down-regulated genes identified by meta-analysis:** (A) T1DKD datasets. (B) T2DKD datasets.

**
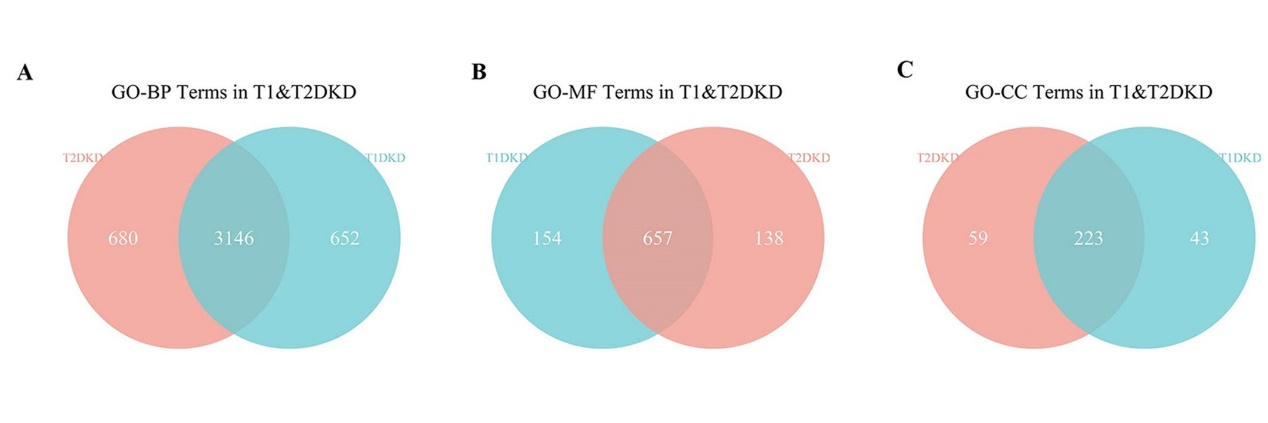
**

**Supplementary Figure 4 Venn diagrams of all significant gene ontology common to T2DKD and T1DKD.**

**
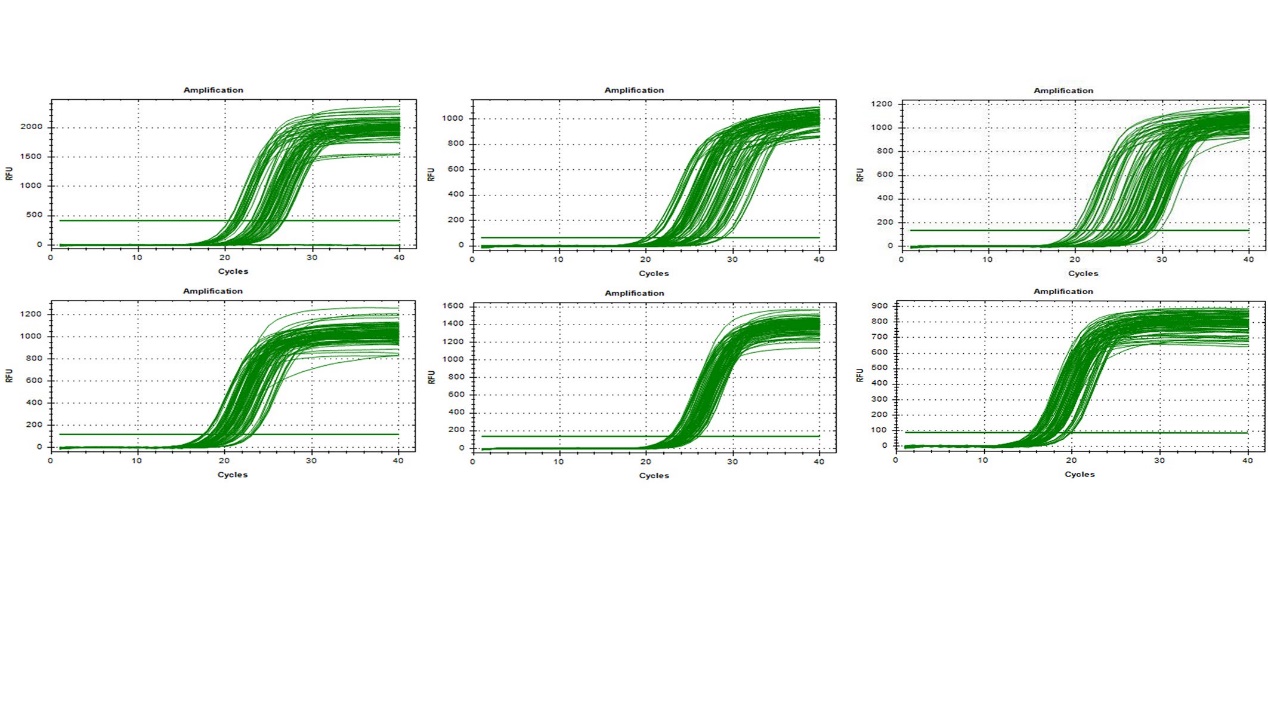
**

**Supplementary Figure 5 Fluorescence plots of qRT-PCR experiments**

**Supplementary Table 1 The top 5 DEGs in TIDKD found in Meta-analysis**

| Up-regulated DEGs | | | | | |
| --- | --- | --- | --- | --- | --- |
| Ranking | Combined P value | Name | | Function | Published role in DKD |
| 1 | 0 | | Ccng1 | Associated with G2/M phase arrest in response to DNA damage.  May be an intermediate by which p53 mediates its role as an inhibitor of cellular proliferation | PMID:32016904 |
| 2 | 0 | | Cdkn1a | Cyclin-dependent protein serine/threonine kinase inhibitor activity  Regulate G1 phase of cell cycle | PMID:31545928 |
| 3 | 0 | | Mgmt | A DNA repair protein involved in cellular defense against mutagenesis and toxicity from alkylating agents | NA |
| 4 | 0 | | Bhmt | Catalyze the conversion of betaine and homocysteine to dimethylglycine and methionine | NA |
| 5 | 0 | | Sulf2 | Selectively removes 6-O-sulfate groups from heparan sulfate and modulates the effects of heparan sulfate | PMID: 26764203 |
| Down-regulated DEGs | | | | | |
| Ranking | Combined P value | Name | | Function | Published role in DKD |
| 1 | 0 | | Nrep | May have roles in neural function cellular differentiation  Down-regulates the expression of TGFB1 and TGF-β2 | PMID: 32539181 |
| 2 | 0 | | Skil | Regulate cell growth and differentiation through TGF-β | PMID 28397834 |
| 3 | 0 | | Grtp1 | A GTPase-activating protein | NA |
| 4 | 0 | | Slco1a1 | Mediate the Na^+^-independent transport of organic anions | NA |
| 5 | 0 | | Tnfaip8 | Act as a negative mediator of apoptosis  May play a role in tumor progression Suppresses the TNF-mediated apoptosis | PMID: 20699119 |

**Supplementary Table 2 The top 5 DEGs in T2DKD found in Meta-analysis**

| Up-regulated DEGs | | | | | | |
| --- | --- | --- | --- | --- | --- | --- |
| Ranking | | Combined P value | Name | | Function | Published role in DKD |
| 1 | 0 | | | Spp1 | Major non-collagenous bone protein that binds tightly to hydroxyapatite | PMID： 23085633 |
| 2 | 0 | | | S100a6 | May function in stimulation of Ca^2+^-dependent insulin release, stimulation of prolactin secretion, and exocytosis | NA |
| 3 | 1.07E-13 | | | Cdkn1a | A potent cyclin-dependent kinase inhibitor  Bind to and inhibit the activity of CDK2 or CDK4 | PMID:31545928 |
| 4 | 1.07E-13 | | | Myof | A type II membrane protein that is structurally similar to dysferlin | NA |
| 5 | 1.69E-13 | | | Gpnmb | A type I transmembrane glycoprotein  Involved in growth delay and reduction of metastatic potential | PMID: 22718188 |
| Down-regulated DEGs | | | | | | |
| Ranking | | Combined P value | Name | | Function | Published role in DKD |
| 1 | 0 | | | Slco1a1 | Mediates the Na^+^-independent transport of organic anions | NA |
| 2 | 0 | | | Acsm3 | Catalyze the activation of fatty acids by CoA to produce an acyl-CoA | PMID: 11158418 |
| 3 | 0 | | | Tmigd1 | May control cell-cell adhesion, cell migration and proliferation  Reduce oxidative stress in kidney | NA |
| 4 | 0 | | | Me1 | NADP-dependent enzyme that generates NADPH for fatty acid biosynthesis | NA |
| 5 | 0 | | | Pzp | Highly expressed in late-pregnancy serum. Inhibit the activity of all four classes of proteinases | NA |

**Supplementary Table 5 Differentially expressed genes from T1DKD datasets identified in Meta-analysis**

| DEGs | Gene Symbols |
| --- | --- |
| 633 up-regulated DEGs | Ccng1,Cdkn1a,Mgmt,Bhmt,Sulf2,Ephx1,Slco2a1,Baat,Plk2,Tmem43,Phlda3,Mgst1,Cldn1,Nek6,Tm6sf2,Igfbp1,Sult1a1,Mdm2,Spsb1,Hacl1,Slc2a2,Mab21l3,Txnip,Amt,Lpar2,Aldh1a1,Ftcd,Apoc3,Cebpd,Enpp5,Glrx,Fga,Impa2,Plcd4,Aqp2,Lox,Zmat3,Nrg1,Pdlim2,Cxcl11,Stc1,Abcc2,Alox5,Pdcd4,Ambp,Grem2,Slc41a3,Foxq1,Pck1,Aldoc,Fbp2,Stra6,Fbxo21,Abca8a,Spp1,Cxcl10,Cnksr1,Aen,Wfdc2,Rasd1,Cdk18,Gsto1,Arnt2,Cwh43,Gadd45b,Slc10a2,Pde7b,Hmgcs2,Fgb,Mfn1,Hfe,Gsta3,Rb1,Rpsa,Grem1,Angptl3,Nup210,Thyn1,Atp12a,Gde1,Rps27l,Acot7,Fam13a,S100g,Tbc1d8,Gpt2,Gstm1,Bzw2,Hmcn1,Tm7sf2,Slc16a9,Acer2,Pla2g12a,Bmp1,Atf5,Pik3ip1,Cpn1,Defb1,Eci1,Mapk13,Dhcr7,Nfkbia,Sema3b,Slc51a,Nnt,S100a10,Tspan6,Plin2,Aco1,Gpnmb,Litaf,Ech1,Hadha,Ccl6,Rnf125,S100a6,Per1,Kalrn,Kynu,Slc25a20,Slc34a2,Slc17a4,Rragd,Upb1,Adhfe1,Icam1,Acacb,Cdk8,Gsta4,Rasd2,Cyp2e1,Pmm1,Angptl4,Asrgl1,Havcr1,Vmp1,Ttc39b,Spryd7,Krt8,Slc13a1,Aco2,Gm2a,Arhgap8,Ttpa,Piga,Itgb4,Cnn3,Cryl1,Calb1,Slc15a1,Trappc6a,Sfxn1,Chmp4c,Tagln,Arrdc3,St3gal5,Tmem171,Gramd1c,Bspry,Pde4dip,Pafah1b3,Fibin,Cytip,Dnase1,Man2a1,Irak3,Il17rc,Tmem120a,Creg1,Clu,Glrx3,Chkb,Arhgap11a,Fn1,Aqp4,Glt1d1,Ccnd1,Lztfl1,Ogfod3,Cpt2,Abcc4,Grpel1,Cyp27a1,Cited2,Aprt,Sdc1,Serpinb6a,Trim2,Slc19a2,Abca7,Rps16,Klhl14,Lpp,Spink8,Rab31,Lpin3,Slc41a1,Fbxo32,Nr1i2,Ctsd,Akr1b8,Lcn2,Chchd6,Gdf15,Cebpb,Sptbn2,Vnn1,Itgav,Sparcl1,Slc23a3,Smpdl3b,Atp6v0a4,Hmga1,Slc27a1,Disp1,Sec14l1,Apex1,Gnb1,Slc13a2,Adtrp,Pex16,Decr1,Rnase4,Chaf1b,Epor,Lyplal1,Ckap2,Klf6,Pgrmc1,Slc5a1,Pi4k2b,Fxyd4,Dhrs7,Pla1a,Tmem97,Mthfd1,Pvr,Scara5,Birc3,Zfp462,S1pr1,Gdpd5,Tgfb2,Serf1,Erbb2,Asb11,Vim,Cubn,Ddx58,Csrp1,C8g,Tacc1,Snn,Sema4g,Ctso,Sec14l2,Maob,Abhd6,Slc38a3,Gpr135,Nol3,Nicn1,Bag2,Apmap,Plgrkt,Fam114a1,Pbx4,Clcnka,Ctsf,Ddb2,Cp,Clcf1,Ank1,Ccng2,Adm,Slc5a11,Capg,Arrdc1,Fhl2,Plbd1,Rpl11,Arhgef9,Btg2,Mybl1,Vps36,Acsf2,Tmprss2,Gpd1,Xpc,Fstl3,Ip6k2,Sh3bgrl3,Map1b,Pfkl,Extl1,Gca,Limk2,Nfe2l1,Asb5,Slco4a1,Eif4ebp1,Rpl4,Atp9a,Arhgap9,Smtnl2,Lbp,Jade1,Mad2l2,Ola1,Me2,Nxpe4,Pla2g15,Slc43a2,Rhpn2,Anxa3,Lrrc51,Vamp5,Gstz1,Klf11,Rras2,Inhbb,Mmp2,Ceacam1,Rdh11,Rab11fip1,Rps6ka2,Etnk1,Nampt,Bcap29,Il33,Cacnb3,Fblim1,Gab2,Sh3bp4,Gpx4,Rnf149,Cpt1c,Asns,Tpd52,Mif,Pacsin1,Sirt3,Aldh1b1,Acaa2,Slc48a1,Ddb1,Tgif1,Gjb2,Arg2,Dusp5,Mrps9,Ell,Brca1,Adh1,Xdh,Blvra,Pex14,Scg5,Tox3,Casp3,Tfcp2,Pigr,Slc27a2,Zfp956,Dmrt2,Bmp6,Ndrg4,Clpx,Pde1c,Cirbp,Atp1b1,Abhd12,Rps5,Rasa3,Nop16,Hrct1,Avpi1,Slc17a3,Impact,Dpp4,Akr1c12,Cnppd1,Abcc3,Ddhd2,Spag5,Pcyox1,Tmed6,Cyp26b1,Il17re,Glud1,Mpzl2,Lgals3,Akap7,Zfp385a,Polr2e,Klf15,Rnd2,Slc18b1,F2rl1,Ptgis,Golm1,Thop1,Adgrf5,Trem2,Nipal2,Nthl1,Dgka,Cxcr4,Alkbh3,Epcam,Lgals1,Susd6,Mthfd2,Ppp1r15a,Rarres2,Iqgap2,Tinagl1,Hsdl2,Pced1b,Nadk2,Hcn2,Slc17a1,Hip1r,Alpl,Sgpl1,Wdfy1,Rnf144b,Masp2,Ddc,Pygb,Slc4a1,Crim1,Thsd4,Rpl35,Rbp4,Pear1,Lama5,Adamtsl4,Cidec,Acp5,Atg12,Piezo1,Aqp6,Ugp2,Rps8,Etfdh,Hmgcl,Trpm8,Naga,Rps11,Cd276,Bmp3,Gria3,Epb41l5,Unc5b,Pam16,C3,Agt,Rack1,Cck,Serpinb1a,Hsd17b10,Zcchc24,Tspo,Foxp1,Nqo1,Hivep3,Mbl1,Itm2b,Mfsd4a,Acsl1,Cmtm7,Ncl,Olfm1,Map4k4,Slc39a14,Blvrb,Masp1,Mical3,B2m,Zwilch,Pbxip1,Dkk3,Trap1,Prps1,Dctpp1,Serpinf1,Steap1,Cdc42ep4,Serpine2,Kit,Amacr,Gas6,Nectin2,Kmo,Tor3a,Gamt,Coro2a,Shmt1,Ggta1,Cygb,Rpl18a,Kctd1,Ifit2,Egr1,Rps28,Ccdc3,Vat1,Ssbp4,Slc2a9,Rap1gap2,Iscu,Wdr91,Igf2bp2,Lgals3bp,Adamts4,Gabarapl1,Ica1,Calr3,Dedd2,Stat3,Akip1,Fdft1,Eci2,Ankrd28,Ppa1,Tns2,Pir,Pnrc1,Acsl5,Car2,Rab27a,Ddit4,Tshz2,Aldh1l1,Ifnlr1,Ano10,Tmem9,Prr15,Smyd2,Nid2,Spred3,Krt19,Gchfr,Thbs3,Ncapg2,Eps8l2,Srxn1,Fetub,Anxa5,Dag1,Rab15,Slc39a6,Col8a1,Cyp39a1,Sdcbp,Nfkbiz,Sdk1,Rpl23a,Mcl1,Myl12a,Plec,Arl4a,Tnfrsf1b,Igf1r,Ifi27,Diaph1,Ercc1,Taf4b,Hic2,Rcan2,Chn2,Tspan2,Tlk2,Sfxn3,St6galnac4,Adck5,Tnfaip3,Esam,Itgb2,Pdk1,Nrbp2,Myc,Ppfibp1,Bhlhe41,Cyb5a,Guca2a,Rpl27a,Mal,Aqp8,Anxa4,Pmvk,Slc35d2,Gltp,Fkbp14,Atg16l2,Pcolce,Slc16a1,Ankrd37,Tmigd1,Tbc1d17,Ric3,Antxr1,Ptprr,Hadhb,Cemip,Sacs,Rhod,Gkap1,Zfp385b,Psmd9,Prune2,Acot2,Gstm2,Optn,Rcn1,Glmn,Mex3a,Aatf,Cir1,Rchy1,Cnnm4,Cd82,Lonp2,Abca5,Pam,Ccdc115,Tmem116,Atp2b4,Slc22a7,Ung,Rgs2,Gls. |
| 620 down-regulated DEGs | Bhlhe40,Pmepa1,Igfbp4,Mpv17l,Mthfr,Celsr2,Tnfaip8,Slco1a1,Grtp1,Skil,Nrep,Dnajc22,Cndp1,Pllp,Rgn,Ldhd,Ablim3,Fam151a,Igsf11,Tmem37,Gas2,Pzp,Proc,Kap,Prkd3,Camk2n1,Smad7,Chd3,Rnaseh2b,Fads2,Htra1,Dio1,Galnt14,Coq10b,Rgl1,Tmem86a,Mpdz,Paqr7,Hspbap1,Pecr,Dnaja4,Krt7,Slc35a1,Cldn10,Prlr,Trim35,Gpc4,Hsph1,Ulk2,Serpinf2,Cmip,Myo5a,Slc16a12,Trabd2b,Slc16a13,Pter,Col4a4,Mylk3,Oxct1,Slc22a17,Cnot2,Clk2,Evc,Exoc6,Hnmt,Cbs,Acad10,Clcn6,Tspan5,Timp3,Dclk3,Iah1,Picalm,Angpt1,Slc7a13,Ackr3,Palm,Dock8,Chordc1,Fkbp4,Clcn4,Lactb2,Cpm,Acsl3,Evl,Irf3,Lims2,Esr1,Nr2f6,Kcne1,Kctd17,Dnaja1,Mlph,Parp16,Trib1,Tcn2,Ehd1,Cdr2,Slc11a2,Tnfrsf14,Zfp518b,Mfap3l,Me1,Fam20c,Mpc1,Dmtn,Pura,Capn6,Slco1a6,Ncbp1,Psph,Slc25a13,Ppm1h,Slc22a2,Fmnl1,Gcnt1,Nkd2,Wscd1,Cgnl1,Fahd1,Epha4,Ptgds,Acat1,Scly,Atp11a,Socs5,Adap2,Klhl9,Map3k7cl,Isoc2b,Bdh1,Dnajb5,Hnf1b,Plekha6,Smarca2,Spsb3,Cndp2,Fgf13,Hpcal1,Bmf,Gfra1,Wdr81,Rasa2,Reps2,Nat8,Cux1,Arvcf,Manf,Bloc1s1,Phtf1,Far1,Zhx2,Sat2,Mlec,Slc13a3,Tmem39b,Lrp3,Enpp1,Trib2,Ddr2,Nav2,Slc39a10,Sdf2l1,Ocel1,Egln1,Nuak2,Paqr9,Slc25a35,Sema5a,Neto2,Ahcyl2,Sfrp2,Cyba,Cpxm1,Camta2,Zbtb20,Osgin1,Scd2,Gcgr,Spns2,Pitpnc1,Khdrbs3,Npr3,Atrnl1,Rnpepl1,Opa3,Ass1,Ptms,Efna5,Prdm1,Cx3cl1,B4galt5,Ncor2,Slc22a23,Zc3hav1l,Grb10,Rabggta,Dpp7,C1qtnf3,Hykk,Vwf,Agtrap,Amot,Tmem79,Nrip1,Arhgef15,Tspan18,Gnpda1,Hspa12a,Slc52a3,Arhgap17,Trps1,Hao2,Klhl8,Prkaa2,Met,Coa7,Dgkb,Mapk8ip1,Cyp27b1,Id2,Dab2,Psat1,Amn,Tug1,Zgpat,Lrp2,Vil1,Zmynd15,Bcl6b,Irf2bpl,Fbxo9,Otub2,Slc25a45,Ak4,Nid1,Ttc36,Mycbp,Agtpbp1,Pdp2,Qprt,Ly6e,Ptpdc1,Pdk3,Ppm1m,Rab3il1,Ptn,Cmtm6,Baz1b,Pcyt2,Vldlr,Spats2l,Srd5a2,Csrp2,Gna12,Klf7,Pah,Slc47a1,Il11ra1,Esm1,Emid1,Entpd2,Ap5b1,Tgfbrap1,Tbx2,Arhgap10,Dock3,Prom1,Fam189b,Efhc2,Hexim1,Miox,Hspa5,Sema6d,Snx6,Bcl7b,P4ha1,Csad,Tmem150a,Ubiad1,Rab29,Cdh2,Dcxr,Gcnt2,Gda,Cabp1,Kcnt1,Nectin3,Creld2,Nox4,Mapk4,Tcf4,Eno1,Cgrrf1,Ipp,Mex3b,Samd8,Sugct,Fn3k,Gc,Tbc1d16,Nat8f3,Dusp7,Myo7a,Xbp1,Edem1,Sufu,Dgkg,Cmpk2,Twf1,Phgdh,Lamc3,Umod,Pomk,Abracl,Kbtbd3,Uaca,Slc34a1,Flrt1,Arsb,Synrg,Fmo1,Zbtb40,Igsf3,Fasn,Ggct,Igfbp5,Lmo4,Trim46,B3galt5,Per2,Bckdk,Agxt2,Inpp5f,Aspg,Pnpla3,Xpr1,Rbm4b,Swap70,Prss8,Megf11,Mkks,Hnf4a,Gsap,Tmem80,Mkrn2os,Ldlrad4,Slc39a8,Prr5,Slc25a30,Secisbp2l,Sema4a,Dnajb1,P2ry1,Gosr2,Tmem41b,Lrrc8d,Kctd15,Sh3tc1,Hagh,Fhl1,Cxxc5,Calml4,Faap100,Krt10,Dpy19l3,Odc1,Tulp3,Fads1,Mark1,Rac3,Preb,Pdzk1,Kl,Atp2a2,Pdia4,Bnc2,Ppic,Itpk1,Tmem252,Tmem64,Acsm2,Nudt12,Grhpr,Sned1,Kcnk5,Ccdc6,Traf4,Parp11,Etv6,Slc12a5,Hmgcr,Ces1d,Gba,Prep,Bcl9l,Inmt,Hsd11b2,Tbccd1,Kdr,Skida1,Rnf145,Eml6,Mgat3,Snhg11,Trim59,Dpys,Dnajc12,Agps,Rcc1,Slc25a15,Fggy,Snta1,Ppp1r1b,Abhd14a,Tinag,Elovl1,Atxn7l3b,Ivd,Fmo5,Xpnpep3,Gmppb,Cxcl16,Robo3,Galnt3,Tmem120b,Fam149a,Snap29,Rnft1,Pitpnm1,Caprin1,Sys1,Mmp15,Osbpl3,Fam81a,Acsm1,Cpe,Cryz,Fcgrt,Vegfa,Degs1,Sil1,Fam120b,Etv1,Map3k14,Fchsd2,Piezo2,Stat5a,Tgm2,Rassf2,Acy1,Gatm,Srebf1,Ppard,Fxyd2,Bend7,Prpsap1,Klk1,Ggh,Pi4ka,Nt5e,Polr2b,Rabl2,Akr1b10,Cyp2s1,Cdon,Bcl7a,Lpl,Zfp341,Nr3c1,Nrp1,Ralyl,Fabp5,Slc5a2,Txlng,Phf23,Pdia3,Chrac1,Degs2,Stip1,Neurl4,Hgf,Bphl,Mturn,Mpped2,Emcn,Agtr1a,Samd1,Fmo3,Spats2,Ghr,Eif1b,Fam78a,Mpp6,Etv5,Mtmr10,Coasy,Thap4,Cacna1h,Timm9,Dnmt3a,Inpp5j,Smco4,Lbr,Idi1,Eefsec,Acly,Pdzd2,Srebf2,Blmh,Glb1,Sipa1l1,Plekhb1,Papss1,Gab1,Dpf3,Atp6v0d1,Gga3,Socs7,Slc12a7,Kcnj12,Nectin1,Tatdn2,Ptprq,Kdm1b,Bsnd,Myo10,Tm2d3,Trim47,Igf1,Il10rb,Wdr4,Mx2,Lats2,Osr2,Slc26a4,Ppt2,Rasl10b,Phldb2,Dennd3,Plcb1,C2cd2l,Rara,Oaz2,Klk1b3,Tspan33,Snap91,Smtn,Aplnr,Bag3,Slc9a8,Fhit,Sertad1,Ssx2ip,Pdia5,Igsf10,Ggt1,Klhl36,Armc6,Hspe1,Arfip1,Nt5dc2,Clcn5,Fhod3,Dach1,Vill,Azgp1,Park7,Slc39a3,Stk4,Ppp6r3,Melk,Hacd2,Plscr2,Thg1l,Mkrn1,Sod1,Srp54a,Aspdh,Ngef,Fras1,Mtr,Lpar3,Phkb,Pcbd1,Tmem106c,Tns1,Ubash3b,Rgs3,Trnau1ap,Ten1,Dhrs1,Tmem174,Cyp4b1,Znrf1,Itga6,Dnajc6,Rwdd2b,Pgpep1,Itih1,Plcl1,Colec11,Josd2,Cyb5r4,Arhgef19,Tpmt,Mageh1,Glyctk,Zfp623,Tmbim6,Card10,St8sia1,Creb1,Pknox1,Naglu,Amd1,Bri3bp,Arhgap22,Ccdc163,Slc25a23. |

**Supplementary Table 6 Differentially expressed genes from T2DKD datasets identified in Meta-analysis**

| DEGs | Gene Symbols |
| --- | --- |
| 806 up-regulated DEGs | Spp1,S100a6,Cdkn1a,Myof,Gpnmb,Mki67,Tnfrsf12a,Havcr1,Tifa,Aoc1,Arhgap11a,Birc3,Cdh6,Itgb7,Col8a1,Ubd,Nupr1,Kif20a,Plekho1,Lcn2,Sprr1a,Lox,Tacstd2,Prc1,Lgals3,Trem2,Cp,Timp1,Mab21l3,Ifi27l2b,Hmgcs2,Ltbp2,Clu,Wfdc2,Dclk1,Ccl6,Tnfaip3,Ube2c,Crlf1,Lyz2,Crabp1,Ctsz,Socs3,Cdc20,Arrdc4,Clec10a,Trpv5,Sfn,Tnfaip8l2,Postn,Vim,Asf1b,Cxcl13,Fn1,Krt8,Tacc3,Ezh2,Tpx2,Mmp2,Ucp2,Runx1,Fgb,Rasa4,Krt19,S100a10,Cdkn2b,Slpi,Cenpf,Serpine2,Ncf1,Il24,Capg,Anxa2,Gdpd5,Sulf2,Lamc2,Adora1,Mcm5,Ncaph,Myo1f,Tubb6,Cox6b2,Adra1d,Mybpc2,Ltc4s,Angptl4,Cdk1,Smtnl2,Irf8,Pla2g7,Lsp1,Tagln2,Gc,Ccna2,Klf6,Pld4,C6,Tlr2,Dpysl3,Icam1,Tmem176a,Ramp1,Tnfsf13b,Serpinb8,Tgfb2,Psmb10,Clcf1,Arl5c,Coro1a,Jak3,Hck,Scara5,Arhgap30,Ccl2,Kif22,Slc25a24,Dapp1,Aurkb,Fxyd5,Glrx,Siglec1,S100a4,Brca2,Map3k1,Traf1,Itgb4,Bzw2,Uhrf1,Blnk,C1qb,Axl,Lif,Osmr,Ndc80,Ccr1,Vav1,Sec14l2,Plin3,Glp1r,Sh2d4a,Cspg4,Il19,Nfkb2,Cdkn3,Nek6,Nfkbiz,Col1a1,Kctd1,Gja1,Bcat1,Steap1,Cd276,Bmp1,Sash3,Gstm1,Htra3,Lgals3bp,Mfsd12,Lxn,Plk1,S100a9,Syk,Map1b,Rgs19,Eif4ebp1,Fcer1g,Msr1,Plec,Fgr,Pfkp,Ephx1,Slfn2,Smc2,Cdca7,Rhbdf2,Inf2,Adgrg3,Stat3,Tubb2b,Mcm3,Chrnb1,Hcls1,Fblim1,Rac2,Itgb2,Il1rn,Agmo,Chaf1b,Tspan1,Dkk3,Gabrp,Schip1,F2rl1,Fstl3,Cfp,Tsc22d1,Gria3,Cst7,Cd44,Wdfy4,Pbk,Lgals1,Adgrg6,Mgp,Ccdc88b,Egr2,Tpbg,Micall2,Gas7,Cd74,Pdlim7,Adcy7,Elf3,Cd68,Mmp7,Spc24,Psmb8,Antxr1,Des,C1qa,Zbp1,Sh3bgrl3,Fst,Plekhg6,Ppl,Tap1,Cldn4,Isyna1,Alox5ap,Trpv6,Syt11,Map4k1,Tspan6,Vat1,Sdcbp,Pdpn,Rab30,Tnfrsf1b,Hfe,Stab1,Adamts1,Emilin1,Col6a1,Tapbpl,Pdlim4,Maob,Mthfd1l,Plin2,Itk,Ncf4,Cpne8,Relb,Fgg,Ssbp4,Ifi27,Fes,Eda2r,Layn,P2ry2,Pycard,Adra2a,Plp2,Klra2,Pik3cd,Slc6a12,Cadm4,Enc1,Serpine1,Slc11a1,Rgs14,Akr1c13,Setd7,Matk,Rgs10,Col6a2,Tnfrsf1a,Sh3bp4,Mcm4,Gzmm,Ctse,Cd3d,Cyp27b1,Mthfd2,Anxa5,Thbs1,Unc93b1,Arhgap25,Klf4,Col4a2,Stap1,Emp3,Tead2,Serpinb9,Slc7a6,Ckb,Prnp,Gpr171,Rrm1,Arid5a,Col4a1,Cyp17a1,Naaa,Krt7,Ube2l6,Scrn1,C5ar1,Btg2,Tgif1,Irf1,Bcl3,Sbno2,Ctsw,Col18a1,Ubtd2,Art4,P2ry6,Tor3a,Ctsd,Cd22,Dkk2,Gpr132,Abcc3,Myc,Ebpl,Parp14,Casp12,Cxcl10,Itgal,Mvp,Fetub,Irf7,Pkp1,Plaur,Myom2,Uchl1,Laptm5,Apol9a,Dsn1,Tcirg1,Nfkbie,Plek,Itgae,F2r,Lhfpl2,Rgs16,Cd9,Fam167a,Pdlim1,Mmp14,Parp10,Atp12a,Dhrs7,Tjp3,Was,Dclre1b,Iqgap3,Col3a1,Zap70,Kif21b,Arntl2,Serpinb6b,Slc34a2,Lrrfip1,Dynlrb2,Slc14a1,Cd101,Il18r1,Dtx4,Alox5,Reg3b,Spc25,C1qc,Tmem176b,Casp4,Bub1b,Apoe,Fbn1,Ptpn7,Grem1,Cd3e,A3galt2,Cd5,Ripk3,Dlgap5,Defb11,Tmsb4x,Ccr5,Anxa3,Rasal3,Hk3,Thbs2,Tnfsf8,Dynll1,Agap2,Pcolce,Fbln1,Elmod1,Kcnn4,Impdh1,Tspan8,Edn1,Ctss,Map3k8,Gmip,Aspm,Ctsk,Tmem43,Fermt1,Tmem59l,Numbl,Pmm1,Snx20,Cygb,Psme1,Card14,Cd2,Limk1,Slc2a2,Maged2,Tex10,Angptl7,Ifitm3,Samd3,Cd6,Dsp,Vcam1,Fbp2,Krt23,Arl11,Arrb2,Mdc1,Adamts8,Fbxo2,Tnfrsf9,Tbc1d10c,Tenm4,Mlkl,Sh2b2,Rab8b,Trim6,Tnn,Slc35g2,Gas2l3,Plxnc1,Zc3hav1,Nckap1l,Lcp1,Relt,Angptl3,Capn2,Tubb3,Cdh11,Vtcn1,Zc3h12d,Mrc1,Ano9,Lrp8,Hddc2,Slc15a3,Spi1,Sox9,Csf2ra,Cyp1b1,Cybb,Oas1a,Efemp2,Mmp19,Pde4b,Atp4a,Fga,Dtl,Rab7b,Lck,Gsto1,Gbp2,Igtp,Cd53,Glipr2,Clec4a1,Pld2,Mybl2,Synj2,Pik3r5,Unc13d,Cerk,Col24a1,Heph,Atad2,Arhgap19,Oasl2,Plk2,Ptgfrn,Tpm1,Acap1,Ptgs1,Arhgdib,Slamf6,Cldn1,S1pr2,Atf3,Litaf,Krt17,Dok2,Cxcl1,Pgm2l1,Slc28a2,Tnfsf15,Anln,Aurka,Slamf9,Flot1,Tgfb1,Reg3g,Ctnnd2,Sorcs2,Oas2,Tm6sf1,Adgrg5,Fermt3,Cd37,Apobec1,Capn5,Pdgfb,Podnl1,Cdc25b,Nfkbia,Cnn2,Csf1r,Cchcr1,Cxcr3,Abhd6,Ptprs,Bcl11b,Irak4,Nlrp3,Hsd3b7,Cnp,Cd84,Xpnpep2,Kynu,Def6,Slc8a1,Batf,Irf9,Abca1,Tnfaip8l3,Tnip2,Fcnb,Ncapg2,Ccr6,Klf5,Chek2,Lrat,Slc4a7,Gpr84,Col5a1,Lrfn4,Kif23,Map7d1,Rasd2,Gnai2,Tacc1,Ccdc120,Dnah5,Slc38a1,Dok1,Cmtm3,Prim1,Csf3r,Lig1,Frk,Qsox1,Pim1,Acsf2,Stat2,Scube1,Pid1,Lyn,Ap1s2,Sparc,Lrg1,Map4k4,Espl1,Rasgrp1,Tmem229b,Serpinh1,Fstl1,Anxa6,Rgs1,Cx3cr1,Tpm4,Lyl1,Sct,Cldn7,Birc5,Lamb3,Gna15,Irak3,Crip2,Slc1a5,Loxl1,Col1a2,Ttc9,Rrbp1,Ehf,St14,Mpzl2,Pold1,Hid1,Ly86,Tlr4,Crip1,Sfxn3,Specc1,Ppp1r14b,Cdh3,Plod2,Adam8,Cyp4v3,Fgd2,Cass4,Wnt7a,Il17rb,Parp9,Cma1,Cst3,Arhgap36,Elf4,Gpx7,Ltbp3,Pklr,Tnnt2,Sema7a,Inpp5d,Samhd1,Fam83d,Usp18,Kcnk13,Ankrd23,Il17re,St3gal4,Tmbim1,Lpcat2,Col16a1,Cdt1,Pik3cg,Rab31,Mkx,St3gal2,Rfc3,Nipal2,Itgax,Fam111a,Lpxn,Kif26b,Ptpn18,Sctr,Ulk4,Isg15,Pstpip1,Col7a1,Cthrc1,Lmo1,Ifngr2,Il2ra,Hspb8,Gnaz,Timp2,Gem,Pnoc,Bcl2l11,Asrgl1,Inhba,Havcr2,Sphk1,Slc39a6,Il1r1,Kif6,Casp3,Mcm6,Plat,Igsf6,Timeless,Ect2,Slc7a1,Pkib,Vasp,Mitd1,Mybl1,Selp,Cntf,Akap12,Tmem145,Arhgef6,Sparcl1,Ldha,Kcnab2,Csrp1,Rasa3,Ch25h,Asns,Cd300lf,Lgals9,Col20a1,Ddhd2,Col5a2,Nhlrc3,Spsb1,Zbtb38,Per3,Uba7,Chtf18,S100a8,Smc4,Pdk1,Gda,Map3k6,Upp1,Adar,Gpr153,Fabp1,Nuf2,Cbr3,Bace2,Adamts12,Stk10,Ces2g,Ankrd1,Apaf1,Tns4,Fhdc1,Dctd,Twist2,Sash1,Clip2,Abca8a,Ly6i,Cdc6,Cenpm,Mycl,Paqr8,C1qtnf1,B3galnt1,Plcd4,Srgap2,S100a13,Pold2,Sell,Mapkapk3,Rhbdl2,Ccl7,Pygb,Nepn,Fas,Igf2bp2,Ksr1,Elovl6,Plxdc1,Vstm2b,Nradd,Mx2,Plcd1,Dock11,Fut10,Tbc1d9,Itgb3,Anxa1,Gpr176,Rin3,Clec4a3,Kdelr3,Gprc5a,Apob,Anxa7,Rasal1,Muc1,Clec3b,Etv4,Slc4a11,Sp110,Aldh3b1,P2ry13,Pik3r1,Prkar1b,Ndn,Mgat4a,Twf2,Hcrtr1,Abcc4,Ifi30,Col5a3,Rad54l,Unc119,Comtd1,Ifnar1,Dok3,Scara3,Trpm2,Tnfrsf25,Msl3l2,Nod1,Folr2,St3gal1,Flot2,Gnat2,Prrg4, |
| 529 down-regulated DEGs | Fmo5,Pzp,Me1,Tmigd1,Acsm3,Slco1a1,Slc7a13,Cckar,Esr1,Slc22a2,Csad,Serpinf2,Amacr,C1qtnf3,Ldhd,Atp11a,Slc22a7,Bdh1,Azgp1,Cyp7b1,Ggct,Tspan5,Tmc3,Chrdl1,Cndp2,Akr1c14,Cym,Aifm3,Slc16a4,Alox15,Anxa13,Lipg,Gclc,Fmo3,Cyp2e1,Vill,Dnah9,Acy1,Nt5e,Cndp1,Cyp8b1,Hykk,Masp2,Col19a1,Ntn1,Odc1,Apoh,Ivd,Pank1,Osbp2,Prok1,Hsd17b2,Ggt1,Mpped1,Slc22a22,Dcst1,Slc3a1,Acy3,Cyp51,Mfsd2a,Mep1b,Itih2,Krt10,Acox2,Chst7,Tst,Cacng5,Ehhadh,Idi1,Mep1a,Degs2,Proz,Acss2,Ccl5,Ccdc6,Cbs,Cmbl,Adhfe1,Anks1b,Sult1c2,Gclm,Lactb2,Pkd2l2,Cpne4,Acat1,Tmem64,Ppic,Entpd5,Cat,Pnliprp1,Ttc39c,Mat2a,Oxct1,Slc39a8,Afmid,Fads2,Snx31,Slc22a12,Aldh8a1,Mpst,Aspg,Srd5a2,Chrm3,Cth,Rxfp2,Tmlhe,Fmo4,Mmp9,Matn1,Rnf24,Iqch,Samd8,Cyp1a1,Gcgr,Cd209a,Ncbp1,Ppm1k,Mpv17l,Cryz,Klf15,Npr3,Slc25a39,Aspdh,B4galt5,Aspa,Smarca2,Slc1a1,Aldh1l1,Slc1a4,Fras1,Atrnl1,Slc25a38,Oacyl,Paqr9,Slc52a3,Casr,Lap3,Sh3bp2,Phlpp2,Aldh6a1,Ttc36,Fxyd6,Fah,Fads1,Klf9,Ddah1,Coasy,Dock3,Serpinb12,Col9a1,Tbx10,Tpmt,Mfap3l,Slc22a18,Ido2,Hao2,Hsd11b1,Galnt11,Glb1,Cldn10,Slco4c1,Otog,Nxpe3,Hsd17b11,Pcdh17,Satb2,Mpp6,Ano2,Sds,B3galt5,Scgb1c1,Aadac,Lrtm2,Mccc2,Slco1a4,Nudt12,Sgk2,Cox15,Dgkg,Slc6a11,Mme,Col4a3,Abhd14a,Aqp11,Ubiad1,Pgpep1,Srebf1,Abat,Fahd1,Smoc1,Tpk1,Hibadh,Rgl1,Dpy19l3,Ephx2,Cisd1,Slc22a17,Cdo1,Mas1,Clpx,Timp3,Mettl7b,Sdr39u1,Lactb,Usp26,Wdr81,Ass1,Igf2bp1,Slc9a8,Htr5b,Agtr1a,Pink1,Pm20d1,Pter,Slc15a2,Ccno,Gstz1,Gpc4,Msh4,Thrb,Glyat,Sat2,Hspd1,Pdzd3,Aadat,Dmtn,Chst11,Pla2g6,Galns,Mtor,Sc5d,Rgn,Pgm3,Bphl,Ahcy,Bhmt2,Cryga,Reep5,Oat,Aldh9a1,Abcc12,Cpe,Pcdh8,Haao,Gss,Slc25a15,Pdilt,Mob3b,Anpep,Xcl1,Hpn,Mmp10,Gucy1b2,Pim3,Ethe1,Stxbp5l,Miox,Stat5a,Dnase1,Acadsb,Hgd,Ppp1r1a,Paqr7,Slc25a30,Slc27a2,Pxmp2,Dhfr,Ppt2,Dlk2,Pappa2,Hnf4a,Gjb2,Etv5,Ctsh,Gpm6a,Slc22a6,Ankar,Pdp2,Abcg2,Susd2,Trib2,Irx3,Tfrc,Acmsd,Slc16a14,Nomo1,Tcerg1l,Slc22a1,Fbxo9,Yod1,Cideb,Tlr9,Npas2,Phyhd1,Tal2,Slc25a48,Pkhd1l1,Gas2,Bend5,A1cf,Trpc5,Apom,Doc2a,Mpped2,Agps,Arsg,Galnt14,Qprt,Slc30a1,Prkd3,Dpys,Slc17a2,Slco1a6,Dpp10,Vwa2,Fbxl21,Pfn4,Myt1l,Ccr7,Khdrbs2,Tas1r3,Fbxo36,Dhtkd1,Hoga1,Acadm,Wwp1,Sfxn5,Scd2,Gtpbp4,Gcm1,Ghrl,Tmem86a,Mrps33,Mpc1,Abcd3,Atp8a2,Atp7b,Ipp,Slco1b2,Fbln7,Chd5,Veph1,Shroom1,Car7,Usp2,Lrrc8b,Slc25a13,Slitrk6,Ghr,Mcf2,Plk5,Zfp385c,Dpep1,Slc6a18,Fdx1,Slc41a2,Vax1,Slc52a2,Pth1r,Slc6a13,Grin2b,Rnf5,Acbd4,Agmat,Sfxn2,Npm2,Exoc6,Slc25a18,Rnf152,Znrf2,Mthfr,Lypla1,Pnkd,Spo11,Asb13,Megf11,Ros1,Ablim3,Ak4,Slc2a4,Hagh,Ddt,Gjb1,Cntfr,Slc46a3,Kmo,Aifm1,Clcn6,Wdtc1,Tnfrsf21,Rbpms2,St3gal3,Sntg2,Rab11fip3,Cmtm6,Emid1,Kl,Lrpap1,Grin2c,Slc17a3,Sdsl,Krt83,Lmtk2,Lhcgr,Fcamr,Mbl1,Tmem70,Grhpr,Dcxr,Fbxl2,Egr4,Stox1,Rims1,Igfbp4,Amer2,Napsa,Igsf11,Dclk3,Acsl3,Cyb5a,Ehd1,Ugdh,Rdh10,Pah,Gdap1l1,Htatip2,Adm2,Osgin1,Kir3dl1,Unc45b,Abcc6,Nit1,Ces2h,Chd3,Epha8,Tbc1d16,Nmnat2,Letm1,Ttbk1,Slc16a12,Acox1,Shmt2,Prps2,Syt1,Erlin1,Kif21a,Pdk3,Fam20c,Sdr9c7,Kcnt1,Grm2,Plau,Gpa33,Tango2,Prr5,Gchfr,Cps1,Tmem144,Psat1,Sun3,Tenm2,Pyroxd2,Adck5,Angpt1,Ptgr2,Kctd17,Trps1,Clybl,Nkg7,Fgf1,Slc16a13,Slc9a3r1,C1galt1,Foxn4,Kcna5,Tff3,Atp2a2,Tysnd1,Neto2,Glul,Pllp,Slc35f1,Nefm,Trim9,Htra1,Egfl6,Suclg2,Ggact,Sphk2,Klk14,Echdc2,Id4,Phkb,Bend3,Snx7,Aldh5a1,Asb14,Sucla2,Kcnma1,Smpd2,Sardh,Neu2,Col6a5,Xpot,Cdk5r2,Srrm3,Glyctk,Mogat1,Klk4,Med14,Kdr,Mccc1,Erlin2,Gfm1,Chrna2,Ankrd61,Cxcl12,Slc25a11,Ahrr,Sucnr1,Sdc2,Aacs,Dhrs4,Tbc1d19,Tlcd2,Pkd2l1,Cacna1f,Cyp2r1,Gnmt. |

**Supplementary Table 7 Shared differentially expressed genes in T1DKD and T2DKD datasets identified in Meta-analysis**

| DEGs | Gene Symbols |
| --- | --- |
| 151 shared up-regulated DEGs | Abca8a,Abcc3,Abcc4,Abhd6,Acsf2,Adck5,Adhfe1,Aldh1l1,Alox5,Amacr,Angptl3,Angptl4,Antxr1,Anxa3,Anxa5,Arhgap11a,Asns,Asrgl1,Atp12a,Birc3,Bmp1,Btg2,Bzw2,Capg,Casp3,Ccl6,Cd276,Cdkn1a,Chaf1b,Clcf1,Cldn1,Clpx,Clu,Col8a1,Cp,Csrp1,Ctsd,Cxcl10,Cyb5a,Cygb,Cyp2e1,Ddhd2,Dhrs7,Dkk3,Dnase1,Eif4ebp1,Ephx1,F2rl1,Fblim1,Fbp2,Fetub,Fga,Fgb,Fn1,Fstl3,Gchfr,Gdpd5,Gjb2,Glrx,Gpnmb,Grem1,Gria3,Gstm1,Gsto1,Gstz1,Havcr1,Hfe,Hmgcs2,Icam1,Ifi27,Igf2bp2,Il17re,Irak3,Itgb2,Itgb4,Kctd1,Klf15,Klf6,Kmo,Krt19,Krt8,Kynu,Lcn2,Lgals1,Lgals3,Lgals3bp,Litaf,Lox,Mab21l3,Maob,Map1b,Map4k4,Masp2,Mbl1,Mmp2,Mpzl2,Mthfd2,Mybl1,Myc,Ncapg2,Nek6,Nfkbia,Nfkbiz,Nipal2,Pcolce,Pdk1,Plcd4,Plec,Plin2,Plk2,Pmm1,Pygb,Rab31,Rasa3,Rasd2,S100a10,S100a6,Scara5,Sdcbp,Sec14l2,Serpine2,Sfxn3,Sh3bgrl3,Sh3bp4,Slc17a3,Slc22a7,Slc27a2,Slc2a2,Slc34a2,Slc39a6,Smtnl2,Sparcl1,Spp1,Spsb1,Ssbp4,Stat3,Steap1,Sulf2,Tacc1,Tgfb2,Tgif1,Tmem43,Tmigd1,Tnfaip3,Tnfrsf1b,Tor3a,Trem2,Tspan6,Vat1,Vim,Wfdc2. |
| 154 shared down-regulated DEGs | Abhd14a,Ablim3,Acat1,Acsl3,Acy1,Agps,Agtr1a,Ak4,Angpt1,Aspdh,Aspg,Ass1,Atp11a,Atp2a2,Atrnl1,Azgp1,B3galt5,B4galt5,Bdh1,Bphl,C1qtnf3,Cbs,Ccdc6,Chd3,Clcn6,Cldn10,Cmtm6,Cndp1,Cndp2,Coasy,Cpe,Cryz,Csad,Cyp27b1,Dclk3,Dcxr,Degs2,Dgkg,Dmtn,Dock3,Dpy19l3,Dpys,Ehd1,Emid1,Esr1,Etv5,Exoc6,Fads1,Fads2,Fahd1,Fam20c,Fbxo9,Fmo3,Fmo5,Fras1,Galnt14,Gas2,Gc,Gcgr,Gda,Ggct,Ggt1,Ghr,Glb1,Glyctk,Gpc4,Grhpr,Hagh,Hao2,Hnf4a,Htra1,Hykk,Idi1,Igfbp4,Igsf11,Ipp,Ivd,Kcnt1,Kctd17,Kdr,Kl,Krt10,Krt7,Lactb2,Ldhd,Me1,Megf11,Mfap3l,Miox,Mpc1,Mpp6,Mpped2,Mpv17l,Mthfr,Mx2,Ncbp1,Neto2,Npr3,Nt5e,Nudt12,Odc1,Osgin1,Oxct1,Pah,Paqr7,Paqr9,Pdk3,Pdp2,Pgpep1,Phkb,Pllp,Ppic,Ppt2,Prkd3,Prr5,Psat1,Pter,Pzp,Qprt,Rgl1,Rgn,Samd8,Sat2,Scd2,Serpinf2,Slc16a12,Slc16a13,Slc22a17,Slc22a2,Slc25a13,Slc25a15,Slc25a30,Slc39a8,Slc52a3,Slc7a13,Slc9a8,Slco1a1,Slco1a6,Smarca2,Srd5a2,Srebf1,Stat5a,Tbc1d16,Timp3,Tmem64,Tmem86a,Tpmt,Trib2,Trps1,Tspan5,Ttc36,Ubiad1,Vill,Wdr81. |

**Supplementary Table 8 Primer sequences used in qRT-PCR experiment**

| Primer name | Sequence (5' to 3') |
| --- | --- |
| rat-IDI1-For | CAGAGATCGGATGCTAAAATTACC |
| rat-IDI1-Rev | CCCAGCTCCGCCTTTAAG |
| rat-ACSL3-For | ATGAAAACGGACAGAGGTGG |
| rat-ACSL3-Rev | TGCCTCAACTTTGCCTAGAG |
| rat-COASY-For | AGAGTCACAAGGAAAACGAGG |
| rat-COASY-Rev | CAGCACATAGATACCTGACGG |
| rat-GRHPR-For | CTCAGGAAGCAGCAGAGTTT |
| rat-GRHPR-Rev | GTGTTAAGGAGCAGGACACTAC |
| rat-FADS2-For | AAGATGCTACGGATGCCTTC |
| rat-FADS2-Rev | TCGGTGATCTGAGAGCTTTTG |
| rat-ASS1-For | CTTGGACCTCTTCATGTACCTG |
| rat-ASS1-Rev | ATGAGCGTGGTAAAGGATGG |
| rat-ACAT1-For | TGCTACACGAACTCCCATTG |
| rat-ACAT1-Rev | ATGACGTTGCCCATGTAGAC |
| rat-MPV17L-For | CTATCCGTGGCCCACTAACG |
| rat-MPV17L-Rev | ATCACCGCTCTGCTGTGAAA |
| rat-HMGCS2-For | CAACTTATACAAGGGTCTAGAGGC |
| rat-HMGCS2-Rev | TGTGGAGAGGTAAAGGGAGG |
| rat-OXCT1-For | AGCCATCACGGGAGACTTTG |
| rat-OXCT1-Rev | GGGCTTACCAGACTTGGCTT |
| rat-BDH1-For | CTACACCAGTCAGGCAGATG |
| rat-BDH1-Rev | AACCCAAATCCAGAGTCACAG |
| rat-β-ACTIN-For | ACGGTCAGGTCATCACTATC |
| rat-β-ACTIN-Rev | GATGCCACAGGATTCCATAC |
